# How does the effectiveness of strategies to improve healthcare provider practices in low-income and middle-income countries change after implementation? Secondary analysis of a systematic review

**DOI:** 10.1136/bmjqs-2020-011717

**Published:** 2021-05-18

**Authors:** Catherine Arsenault, Samantha Y Rowe, Dennis Ross-Degnan, David H Peters, Sanam Roder-DeWan, Margaret E Kruk, Alexander K Rowe

**Affiliations:** 1 Department of Global Health and Population, Harvard T H Chan School of Public Health, Boston, Massachusetts, USA; 2 CDC Foundation, Atlanta, Georgia, USA; 3 Harvard Medical School, Boston, Massachusetts, USA; 4 Harvard Pilgrim Health Care Institute, Boston, Massachusetts, USA; 5 Department of International Health, Johns Hopkins University Bloomberg School of Public Health, Baltimore, Maryland, USA; 6 Department of Health Systems, Impact Evaluation and Policy, Ifakara Health Institute, Dar es Salaam, United Republic of Tanzania; 7 Malaria Branch, Division of Parasitic Diseases and Malaria, Center for Global Health, US Centers for Disease Control and Prevention, Atlanta, Georgia, USA

**Keywords:** health services research, implementation science, performance measures, quality improvement, standards of care

## Abstract

**Background:**

A recent systematic review evaluated the effectiveness of strategies to improve healthcare provider (HCP) performance in low-income and middle-income countries. The review identified strategies with varying effects, including in-service training, supervision and group problem-solving. However, whether their effectiveness changed over time remained unclear. In particular, understanding whether effects decay over time is crucial to improve sustainability.

**Methods:**

We conducted a secondary analysis of data from the aforementioned review to explore associations between time and effectiveness. We calculated effect sizes (defined as percentage-point (%-point) changes) for HCP practice outcomes (eg, percentage of patients correctly treated) at each follow-up time point after the strategy was implemented. We estimated the association between time and effectiveness using random-intercept linear regression models with time-specific effect sizes clustered within studies and adjusted for baseline performance.

**Results:**

The primary analysis included 37 studies, and a sensitivity analysis included 77 additional studies. For training, every additional month of follow-up was associated with a 0.19 %-point decrease in effectiveness (95% CI: –0.36 to –0.03). For training combined with supervision, every additional month was associated with a 0.40 %-point decrease in effectiveness (95% CI: –0.68 to –0.12). Time trend results for supervision were inconclusive. For group problem-solving alone, time was positively associated with effectiveness, with a 0.50 %-point increase in effect per month (95% CI: 0.37 to 0.64). Group problem-solving combined with training was associated with large improvements, and its effect was not associated with time.

**Conclusions:**

Time trends in the effectiveness of different strategies to improve HCP practices vary among strategies. Programmes relying solely on in-service training might need periodical refresher training or, better still, consider combining training with group problem-solving. Although more high-quality research is needed, these results, which are important for decision-makers as they choose which strategies to use, underscore the utility of studies with multiple post-implementation measurements so sustainability of the impact on HCP practices can be assessed.

## Introduction

A competent health workforce is essential to produce better health outcomes. However, wide deficits in the performance of healthcare providers (HCPs; such as physicians, nurses or midwives) have been documented in low/middle-income countries (LMICs), with poor adherence to evidence-based standards of care being common.^1^ Studies on a range of health conditions have shown that HCPs typically provide less than half of recommended care, consultations are short, safety concerns are common and diagnoses are frequently incorrect.^1 2^ Inadequate quality of care has many causes, such as poor health worker knowledge, motivation and support systems; insufficient financing, leadership and information systems; and a lack of essential medicines, vaccines and equipment.^1^ This widespread evidence on poor quality has led to an increased attention to strategies that can improve HCP performance in LMICs.

Many strategies to improve HCP performance in LMICs have been tested, some of whose effects have been summarised in systematic reviews.^3–14^ In this report, the term ‘strategy’ includes any intervention that could plausibly improve HCP performance either directly (eg, training or HCP incentives) or indirectly, by changing an HCP’s physical, economic or policy environment (eg, providing medicines, reducing user fees or implementing new health regulations).^15^ The recently published Health Care Provider Performance Review (HCPPR),^16^ the most comprehensive systematic review on this topic, aimed to assess the effectiveness of all strategies tested in LMICs with an eligible study design and identify strategy characteristics associated with increased effectiveness.^17 18^

A previous analysis of HCPPR data revealed that several strategies appeared to improve a key aspect of HCP performance, the quality of HCP practices (eg, correct patient diagnosis and treatment),^16^ which corresponds to the Institute of Medicine’s definition of healthcare quality.^19^ For example, for professional HCPs (generally, facility-based health workers), training or supervision alone typically had moderate improvements on HCP practices (median of 10.3 and 14.8 percentage-points (%-points), respectively), combining training and supervision had somewhat larger effects (18.0 %-points), and group problem-solving alone and group problem-solving combined with training had moderate to large improvements (28.0 and 56.0 %-points, respectively). This previous analysis evaluated a strategy’s effect at only one point in time—the absolute %-point change in an HCP practice outcome between baseline and the last follow-up measurement in each study. However, from a public health or programmatic perspective, a strategy’s effect is conceptually the product of the magnitude of the strategy’s effect and its duration. Whether these strategies’ beneficial effects tended to be sustained, or wane, over time remained unclear.

We conducted a secondary analysis of HCPPR data to explore how strategy effectiveness changed over time. Our findings should be relevant to anyone involved in improving healthcare quality in LMICs. Programme managers, funders, technical agencies and other development partners could use these results to help select new strategies, or refine current ones, that are more likely to have a lasting impact on HCP practices and quality of care over time.

## Methods

### Data source: the HCPPR

We analysed data from the HCPPR, which included both published and unpublished studies from the 1960s to 2016 that quantitatively evaluated strategies to improve HCP performance. The literature search included 52 electronic databases for published studies and 58 document inventories for unpublished studies. HCPs were broadly defined as hospital-based, clinic-based or community-based health workers, pharmacists and shopkeepers who sell medicines. Public and private sector HCPs were included. Eligible study designs were controlled before–after studies, post-intervention-only studies with a randomised comparison group, and interrupted time series (ITS) studies with at least three baseline and follow-up measurements. Studies on any health condition were included, in any language. There were no restrictions on the type of strategy, as long it had at least one component that could plausibly affect HCP performance. The HCPPR identified 207 unique strategy components. The risk of bias assessment, which was based on guidance from the Cochrane Effective Practice and Organisation of Care Group,^20^ categorised each study as having either low, moderate, high or very high risk of bias according to the following risk of bias domains (some domains only applied to certain study designs): dataset completeness, balance in baseline outcome measurements, balance in baseline characteristics, outcome reliability, adequacy of concealment of allocation, strategy’s likelihood of affecting data collection, strategy’s independence from other changes, and number of data points before and after the strategy’s implementation. The full systematic review methods, including a detailed search strategy, are described elsewhere.^15 16^

### Types of outcomes included

The HCPPR identified continuous outcomes (eg, number of medicines prescribed per patient) and outcomes expressed as percentages (eg, proportion of patients treated correctly). We excluded continuous outcomes because previous analyses revealed inconsistent effects that could be highly variable.^16^ We also restricted the present analysis to HCP practice (or process-of-care) outcomes expressed as percentages. We excluded outcome categories such as patient health outcomes, utilisation of health services and HCP knowledge. Practice outcomes are a central dimension of interest, and improvements in these outcomes have been found to be correlated with improvements in patient health outcomes.^18^ Moreover, the causal pathway between an improvement strategy and processes of care is shorter than for patient health outcomes. Practice outcomes were the most commonly reported across all studies and reflect HCP behaviours and clinical actions such as assessing patients, diagnosing conditions, providing treatments, counselling, referring patients, consultation time and documentation. Studies most frequently collected data on practice outcomes using record reviews, interviews with patients, observations of HCP–patient interactions and interviews with HCPs.^16^ For the majority of studies, reliable outcomes were measured, and the strategy being tested was unlikely to have affected data collection.^15^

### Types of strategies included

Although the HCPPR included strategies aimed at any provider type, we restricted our analysis to strategies primarily focused on health facility-based HCPs. Therefore, four studies that predominantly included lay or community health workers were excluded. Furthermore, we selected strategies that were each tested by at least four study comparisons that each included at least one HCP practice outcome expressed as a percentage. A study comparison signifies a comparison of a group exposed to a new strategy and a control group not exposed to any new strategy (or an active strategy group and its historical control in ITS studies).

Altogether five strategies were included: HCP training alone (ie, without other intervention components), HCP supervision alone, training combined with supervision, group problem-solving alone and group problem-solving combined with training (descriptions in box 1). These strategies could improve HCP practices by either directly impacting individual HCPs (eg, training or supervision) or indirectly, by changing the system in which the HCPs work (eg, group problem-solving). We included all HCPPR-eligible studies for these five strategies, even if the study had only one follow-up measurement; although such studies were only used for sensitivity analyses (see below).

Box 1 **Definition of strategies**

**Training** was defined as an education-based strategy of any duration, academic detailing (ie, one-on-one training by an opinion leader) and informal education of healthcare providers (HCPs) by their peers. Training tended to have a short implementation period (most lasted 1–5 days).

**Interrupted training** was defined as on-the-job training led by a facilitator typically in group settings and whose content was delivered in separate, short sessions spaced over time. Interrupted training does not include HCP self-study, academic detailing, peer-to-peer education, preservice training or refresher training with the same content repeated over time. It also does not include a single-training curriculum that is scaled up to different trainee groups over a long time period. An example of interrupted training is a 4-day training curriculum for a single group of trainees that is separated into four 1-day training sessions (with each session presenting different materials) and implemented by having a different 1-day training session for the trainee group each Monday over 4 weeks.

**Supervision** included any of the following:

- Implementing or revising routine supervision.
- Benchmarking.
- HCPs sought second opinion from peer or higher level HCP.
- HCP received instructions from higher level HCP.
- Managers of HCPs received supervision.
- Managers of HCPs received training.
- HCP received support from non-supervisory staff.
- Audit with in-person feedback.
- Audit with written feedback.
- Monitoring of HCP practice parameters.
- Peer review.
- Health facilities were inspected to monitor for deviations from regulations.
- Drug utilisation review or evaluation.
- Performance appraisal practices.

Supervision was defined as **one time** if all supervision components were conducted only once during the study period. If at least one supervision component was repeated more than one time, supervision was defined as **ongoing**. Supervision had no fixed implementation period and tended to be ongoing.

**Group problem-solving** included continuous quality improvement, improvement collaboratives, and HCPs holding meetings to discuss problems and solutions with or without formal teams. The first rounds of group problem-solving activities typically were completed in 1–3 months.

### Statistical analysis

Our analysis was done in two steps. First, we calculated an effect size for each follow-up time point after a strategy was implemented. Second, regression models were used to assess associations between follow-up time and effect sizes. For discrete strategies such as a single training session, we included time points after the strategy was fully implemented (eg, all training sessions were completed). For ongoing strategies such as monthly supervision or quarterly group problem-solving meetings, we included time points after the first round of the strategy was completed (eg, the first round of monthly supervision completed or first group problem-solving session completed). This specification for ongoing strategies was justified because these strategies often addressed evolving quality-of-care issues, and the specific time point when they had been ‘fully implemented’ was unclear. Time points occurring during strategy implementation were excluded.

#### Calculations of time-specific effect sizes

Each of the five included strategies was evaluated by multiple studies. Each study measured effectiveness using one or more outcomes, and outcomes were measured at one or more time points after the strategy implementation. To estimate changes in strategy effectiveness over time, we calculated effect sizes at each follow-up time point using the time-specific measurements extracted from these studies. For example, if a study provided outcome measurements at 3, 6 and 9 months after strategy implementation, we calculated three effect sizes.

Effect sizes were calculated differently for ITS and non-ITS studies. For non-ITS studies, effect sizes were calculated using a difference-in-differences specification with the following equation (where *t* is the number of months since strategy implementation):

Effect size_t_ = (follow-up_t_–baseline)_strategy arm_–(follow-up_t_–baseline)_control arm_

For ITS studies, we used segmented linear regression models to estimate a summary effect size at each follow-up time point that incorporated both the level and trend effects.^21^ The summary effect size at time *t* was calculated as the observed outcome at time *t* minus a predicted counterfactual that was the outcome based on the pre-intervention trend extended to time *t*. Segmented linear regression modelling for ITS studies was performed using SAS V.9.4.

Effect sizes were calculated as the absolute %-point differences in the outcome and were calculated such that positive values indicated improvement. We first assessed effectiveness trends visually by plotting effect sizes over time for each strategy. In each graph, we used a different colour per study and connected time-specific effect sizes from the same outcome with a line.

#### Regression analyses

Next, we estimated the association between time and strategy effectiveness by regressing time-specific effect sizes on the number of months since the strategy was implemented. We used two-level random-intercept linear regression models with time-specific effect sizes clustered within studies, and SE estimation accounted for clustering at the study level. Because effect sizes tend to be lower when baseline performance is high (because there is less room for improvement),^16^ we also adjusted the models for baseline performance. We acknowledge the potential for confounding by other factors; however, we were hesitant to explore confounding further because of the small sample size of studies included in the primary analysis (see below) and concerns of overspecification of the regression models. The resulting regression coefficients for the time variable represent the mean %-point change in effectiveness per additional month of follow-up. Detailed specifications for the statistical models are described in box A in the online supplemental materials. We used post-estimation commands to predict effect sizes at different follow-up time points and displayed these predicted values in the graphs as a dashed black line, along with the 95% confidence bands around those predicted effect sizes (displayed as grey bands bounded by dashed black lines). We performed an unweighted analysis because most studies had SEs that did not account for the clustered nature of the data (eg, patient observations clustered within health facilities) or had incomplete sample size information that made it challenging to create a reasonably valid analysis weight.

10.1136/bmjqs-2020-011717.supp1 Supplementary data

We defined three categories of studies based on their design and on the length of follow-up: ITS studies (which all had at least three follow-up measurements of the outcome), non-ITS studies with at least two follow-up measurements, and non-ITS studies with only one follow-up measurement. We conducted our primary analysis on two datasets: (1) ITS studies only (the ideal dataset for assessing time trends because studies had the most follow-up measurements, and effect sizes incorporated baseline time trends, but limited because strategies were usually tested by few ITS studies), and (2) ITS plus non-ITS studies with at least two follow-up measurements (considered the ‘best’ dataset because it maximises sample size, and all studies contributed a true time trend). Outcomes that were assessed with just one follow-up measurement were only included in a sensitivity analysis. Regression modelling was only performed if there were at least three studies in a given analysis.

#### Sensitivity analyses

We performed three sensitivity analyses. First, to assess the impact of a few outlier studies with substantially longer follow-up periods, we conducted an analysis of ITS plus non-ITS studies with at least two follow-up measurements using effect sizes up through the latest time point that involved at least three studies. Results reflecting three or more studies might be considered to be more generalisable.^16^ Second, to assess the impact of non-ITS studies with only one follow-up measurement, we analysed a dataset of ITS plus all non-ITS studies. Non-ITS studies with only one follow-up measurement made up the largest proportion of included studies and increased the sample size substantially for regression modelling; however, these studies do not contribute true time trends, and associations between time and strategy effectiveness among them are subject to selection bias. Third, we further stratified the training and supervision strategies according to how they were delivered: all at once or over several sessions. Training was either done at one time or was interrupted, that is, done over two or more separate sessions (sometimes called ‘low-dose, high-frequency training’).^22^ Similarly, supervision strategies were either done once (ie, only one visit during the study period) or were ongoing (if at least one supervision component was repeated more than once).

All random-intercept linear regression modelling was performed using STATA V.16.0. The STATA code, dataset and an associated data dictionary are publicly available and can be found at: https://github.com/catherine-arsenault/Do-files-change-in-effectiveness-HCPPR-2020. Other data issued from the HCPPR can be found at: http://www.HCPperformancereview.org.

## Results

We included 37 studies in the primary analysis and 114 studies in the sensitivity analyses (online supplemental tables A and B). Altogether, there were 121 study comparisons. Most studies had one comparison (ie, an intervention arm vs a control arm), three studies each had two comparisons (ie, two intervention arms and a control arm), and two studies each had three comparisons. The studies were conducted using a variety of study designs in diverse contexts, although studies were more often in public sector settings, outpatient health facilities, from low-income countries and in sub-Saharan Africa (online supplemental table C). Most studies in the primary analysis (89%) were published since 2000, and 65% had a high or very high risk of bias.

The primary analysis included 17 studies of training alone, 5 of supervision alone, 5 of training plus supervision, 8 of group problem-solving alone and 4 of group problem-solving plus training (see table 1 and online supplemental table B). The studies evaluated a varied set of training, supervision and group problem-solving activities. Training alone was mostly group in-service training, supervision alone primarily involved audit with in-person feedback, training plus supervision was mostly group in-service training plus routine supervision, and both group problem-solving alone and group problem-solving plus training primarily involved improvement collaboratives (online supplemental table C).

**Table 1:** Results from mixed-effects linear regression models: time trends in the effectiveness of five strategies*

Analysis	No of studies	No of effect sizes	Latest month of follow-up	Coefficient	P value	95% CI	
**(A) Training alone**							
ITS studies only	2	29	11	NA	NA	NA	NA
i. ITS+non-ITS studies with >2 follow-up measurements	17	192	30	−0.19	0.023	−0.36	−0.03
*Sensitivity analyses*							
ii. ITS+non-ITS studies with >2 follow-up measurements from >3 studies†	16	177	10.5	−0.23	0.228	−0.59	0.14
iii. ITS+all non-ITS studies	65	549	30	−0.35	0.001	−0.56	−0.14
**(B) Supervision alone**							
i. ITS studies only	3	39	33	0.88	<0.0001	0.72	1.03
ii. ITS+non-ITS studies with >2 follow-up measurements	5	47	33	0.86	<0.0001	0.67	1.04
*Sensitivity analyses*							
iii. ITS+non-ITS studies with >2 follow-up measurements from >3 studies†	5	33	6	−0.54	0.652	−2.89	1.81
iv. ITS+all non-ITS studies	16	90	33	0.82	<0.0001	0.58	1.05
**(C) Training plus supervision**							
ITS studies only	0	0	NA	NA	NA	NA	NA
i. ITS+non-ITS studies with >2 follow-up measurements	5	90	16	−0.40	0.005	−0.68	−0.12
*Sensitivity analyses*							
ii. ITS+non-ITS studies with >2 follow-up measurements from >3 studies†	5	82	6	−0.52	0.211	−1.35	0.30
iii. ITS+all non-ITS studies	22	156	16	−0.46	0.200	−1.17	0.25
**(D) Group problem-solving alone**							
i. ITS studies only	8	230	34	0.50	<0.0001	0.37	0.64
ii. ITS+non-ITS studies with >2 follow-up measurements	8	230	34	0.50	<0.0001	0.37	0.64
*Sensitivity analyses*							
iii. ITS+non-ITS studies with >2 follow-up measurements from >3 studies†	8	225	30	0.53	<0.0001	0.39	0.67
iv. ITS+all non-ITS studies	12	245‡	34	0.46	<0.0001	0.31	0.61
**(E) Group problem-solving plus training**							
i. ITS studies only	4	199	24	−0.13	0.485	−0.51	0.24
ii. ITS+non-ITS studies with >2 follow-up measurements	4	199	24	−0.13	0.485	−0.51	0.24
*Sensitivity analyses*							
iii. ITS+non-ITS studies with >2 follow-up measurements from >3 studies†	4	116	12.5	−0.45	0.259	−1.23	0.33
iv. ITS+all non-ITS studies	4	199	24	−0.13	0.485	−0.51	0.24

Roman numerals number each regression analysis performed and are referred to in the STATA code and diagnostic plots on GitHub.

Coefficient is the model coefficient, which is the mean percentage-point change in healthcare provider practice outcomes per month.

*Estimates from random-intercept linear regression models adjusted for healthcare provider baseline performance.

†The sensitivity analysis used the ‘ITS+non-ITS studies with >2 follow-up measurements’ dataset, but only included effect sizes up through the latest follow-up time that involved three or more studies.

‡One effect size was missing baseline performance and was excluded from the regression modelling.

ITS, interrupted time series; NA, not available.

**Figure 1:**
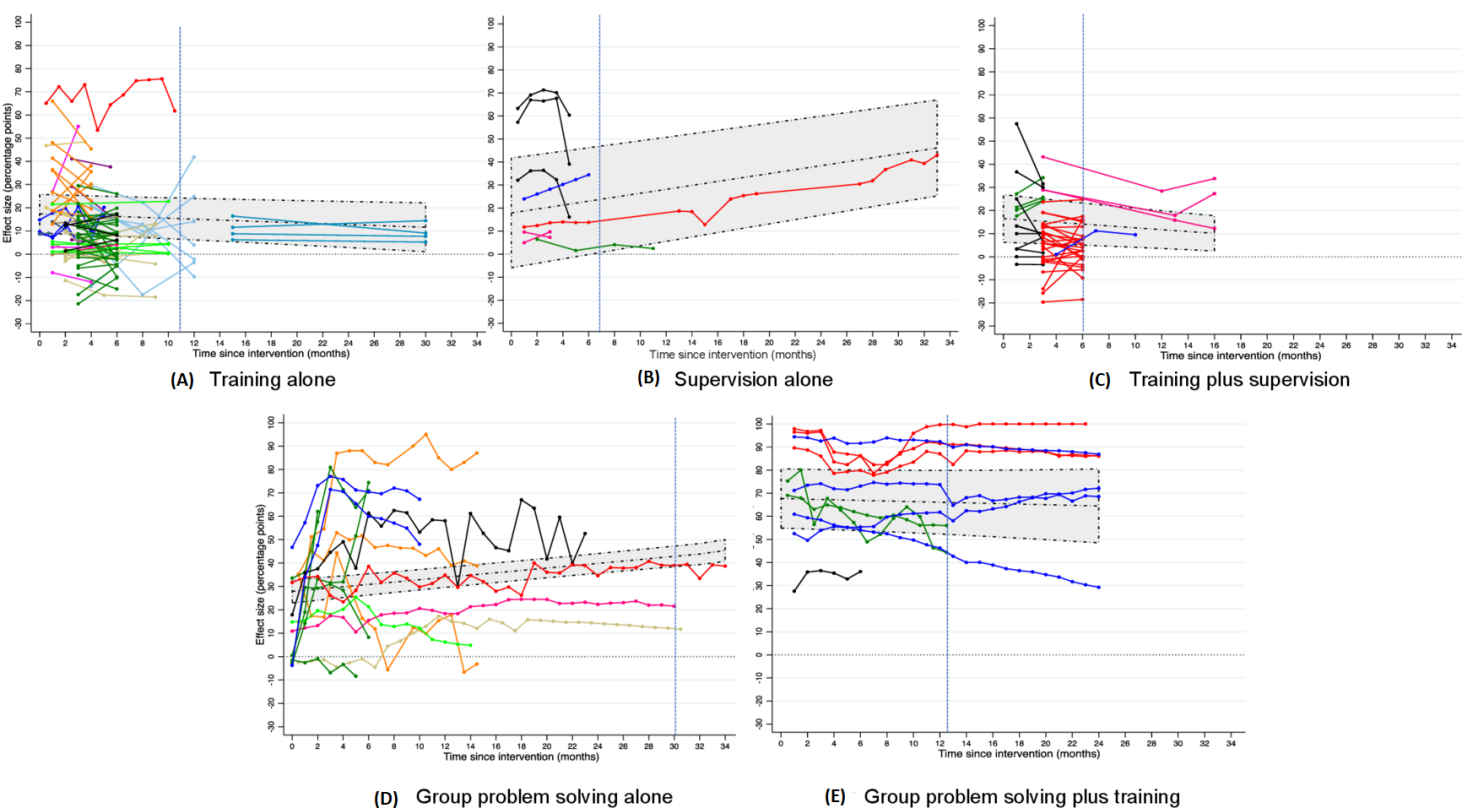
Effectiveness of five strategies over time: (A) training alone, (B) supervision alone, (C) training plus supervision, (D) group problem-solving alone, (E) group problem-solving plus training. Data from all studies with an interrupted time series (ITS) design or non-ITS design with at least two follow-up measurements are shown for each strategy. Each study’s data are represented by one colour, and effect sizes from the same outcome are connected with a line. The middle dashed line in the shaded area indicates the predicted effect size at each time point based on the random-intercept linear regression model with baseline healthcare provider performance held constant at the mean level for all effect sizes for a given strategy (ie, 34.4%, 58.1%, 27.5%, 43.9% and 18.1% for figure 1A–E, respectively). The upper and lower dotted lines at the edges of the shaded area indicate the 95% confidence band around the predicted effect sizes. The blue vertical lines indicate the last follow-up time point involving at least three studies.

Our study included 1240 time-specific effect sizes of 637 practice outcomes (online supplemental table B). The practice outcome categories most commonly measured were treatment, counselling and assessment of patient symptoms (36%, 24% and 16%, respectively; online supplemental table C). Most outcomes (482 or 76%) were assessed using only one follow-up measurement, 121 outcomes (19%) were assessed in a non-ITS study with at least two follow-up measurements, and 34 outcomes (5%) were assessed using an ITS design. Outcomes in ITS studies had between 3 and 35 follow-up measurements per study comparison (median: 24 measurements). figure 1A–E shows the time-specific effect sizes for the five strategies in the primary analysis (ITS and non-ITS studies with at least two follow-up measurements). Figures disaggregated by study design are presented in the online supplemental figures A–E. In the primary analysis, for all strategies combined, the length of follow-up ranged from 1 day to 34 months, with a median of 6 months. Follow-up times differed across strategy types (online supplemental figure F). Mean follow-up time was 5.2 months (range: 0–30 months) for training alone, 8.3 months (0.5–33) for supervision alone, 4.4 months (1–16) for training plus supervision, 10.6 months (0–34) for group problem-solving alone and 11.4 months (0.5–24) for group problem-solving plus training. In the sensitivity analysis of data up through the last time point involving at least three studies, follow-up periods were restricted to 10.5 months for training alone, 6 months for supervision alone, 6 months for training plus supervision, 30 months for group problem-solving alone and 12.5 months for group problem-solving plus training (see blue vertical lines in figure 1A–E).

Table 1A–E shows modelling results for the association between follow-up time and strategy effectiveness, adjusted for baseline performance. For in-service training alone, every additional month of follow-up was associated with a 0.19 %-point reduction (95% CI –0.36 to –0.03) in effectiveness (Table 1A and Figure 1A). Using this model, the predicted improvement achieved just after training (mean of 17.4 %-points at time=0 months) would decline by 13.4% within 12 months. For training plus supervision, follow-up time was also negatively associated with effectiveness with a 0.40 %-point reduction (95% CI –0.68 to –0.12) per month (table 1C). Associations between time and effectiveness for training alone and for training plus supervision were not always statistically significant in sensitivity analyses.

For group problem-solving alone, time was positively associated with effectiveness with a 0.50 %-point increase per month (95% CI 0.37 to 0.64) (table 1D). For group problem-solving plus training, effect sizes tended to be very large immediately after strategy implementation, but follow-up time was not associated with effectiveness (Table 1E and Figure 1E).

Results for the association between time and effectiveness were inconclusive for supervision alone (table 1B). While the primary analyses and one sensitivity analysis found statistically significant positive associations between time and effectiveness for supervision (ranging from a mean of 0.82 to 0.88 %-points per month), another sensitivity analysis showed no association (–0.54 %-points per month; 95% CI –2.89 to 1.81). These findings indicate that the primary results were sensitive to one supervision study with 33 months of follow-up with a strong, positive time trend (see red line in figure 1B), which had a high risk of bias.^16^

The sensitivity analysis that stratified the training and supervision strategies according to how they were delivered (eg, one-time vs interrupted training) found no meaningful differences between the time trends for each strategy strata (online supplemental table E). We question the different trends for the supervision strata because, as previously mentioned, the sensitivity analysis revealed the influence of an outlier study. In general, these stratified results should be interpreted cautiously because several strata had small numbers of studies with at least two follow-up measurements (ie, few included studies contributed true time trend data).

## Discussion

Our objective was to explore how the effectiveness of five strategies to improve HCP practices in LMICs changed over time. If programmes decide to use these strategies, understanding how their effects change over time is critical for designing them to optimise sustainability. To our knowledge, this is the first analysis of data from a systematic review of studies conducted in the LMICs to focus on time trends in strategy effectiveness.

The concept of decay in effectiveness is not unique to strategies in LMICs. Several behavioural interventions to improve HCP practices in high-income countries did not have persistent effects over time.^23–26^

The effect of training alone, a commonly used strategy to improve HCP performance in LMICs,^17^ appeared to decay over time. This result is supported by multiple large observational studies that found training had little to no effect on the quality of primary care services.^27–31^ A separate HCPPR report on training found a larger decline in effect for in-service training alone (0.8–1.0 %-points per month),^17^ however this result was based on a more simplistic analytical approach (only one follow-up measurement per outcome) and thus is probably less valid than our findings. Furthermore, our sensitivity analysis suggested little difference in the effect of training delivered all at once versus over several sessions. Our finding of a negative association between time after training implementation and effectiveness does not necessarily mean that training has low value; rather, training alone might not be sufficient to achieve the desired improvement in HCP practices. Programmes that use training alone might consider periodical refresher training or more impactful training modalities (eg, include clinical practice and training at the HCP’s worksite)^17^ to maintain HCP practices, although a better approach might be to choose a strategy with a larger, more sustained effect.

For supervision alone, another strategy widely used for improving quality in LMICs, we found inconclusive results. Results indicating a positive association between time and effectiveness should be interpreted with caution, as they appeared to be driven by one outlier study with a high risk of bias.^16^ In addition, supervision alone was evaluated by only five studies with mostly short follow-up periods. Multiple large observational studies, which reflect the effect of supervision as implemented at scale by real-world programmes, have found that supervision had no effect on quality.^27 28 31–33^ Similar to our lack of evidence that the effect of one-time supervision was different from ongoing supervision, one previous systematic review of managerial supervision concluded that supervision with more frequent visits was not necessarily more beneficial.^3^ A review of audit and feedback (a type of supervision strategy) in high-income and LMIC settings suggested that audit and feedback might be more effective if it was given by a supervisor or colleague, was provided more than once, delivered in both verbal and written formats, and when it included explicit targets and an action plan.^9^ To better understand time trends of supervision effectiveness, it would be useful to perform meta-analyses to identify attributes of supervision strategies associated with an increasing trend in effectiveness and conduct additional high-quality trials of supervision with longer follow-up periods.

For the strategy combination of training plus supervision, follow-up time also seemed negatively associated with effectiveness, and the magnitude of the negative association was somewhat larger than for training alone. This finding is a reminder that the magnitude or duration of effect of a multifaceted strategy might not be a simple combination of the effects of the individual strategy components. Differences in the effects of a single-component versus multicomponent strategy might be due to studies of each strategy group varying systematically by contextual factors for which we did not adjust in our analyses, such as economic level, quality of infrastructure, provider payment mechanism or adequacy of healthcare financing.

For strategies with group problem-solving, their effects appeared to increase or remain constant over time. Group problem-solving strategies mostly involved continuous quality improvement and improvement collaboratives, which are widely used in high-income countries, with growing use in LMICs.^7 34 35^ *Continuous quality improvement* involves the use of a multidisciplinary team to conduct ongoing cycles of planning, implementing, monitoring and revising quality improvement strategies at one particular healthcare site over time.^7^ An *improvement collaborative* involves teams from multiple sites participating in a structured process to test change ideas using improvement cycles and to share their improvement ideas, experiences and data on performance.^7 35^ The increasing effect might be explained by these cycles leading to a final strategy that is better tailored to overcome obstacles to quality in a specific programmatic context, or perhaps by HCPs being motivated to improve because they are aware that their practices were being monitored. These approaches to problem-solving may be better suited to addressing complex performance obstacles and may help promote more sustained changes in organisational culture, functions and structure. Compared with group problem-solving only, in which several studies found a sharp increase in effectiveness during the first 4 months after implementation, our results for group problem-solving plus training revealed very large effect sizes immediately after implementation. It is plausible that training (perhaps combined with health workers’ awareness of being monitored) caused the short-term boost in HCP practices, which was sustained by group problem-solving.

The positive and sustained time trend findings for strategies involving group problem-solving are encouraging; however, these strategies are more complex to implement. Knowing what factors lead to their successful implementation over time would be extremely helpful. A previous analysis of HCPPR data showed that group problem-solving strategies might be more effective in moderate-resource settings than in low-resource settings.^16^ While other evidence on the determinants of the success of such strategies comes from studies in high-income countries,^35–37^ this evidence might be relevant for LMICs. To improve the effectiveness of continuous quality improvement strategies, the use of clinical process measures, collaboration and communication between HCPs, and having frequent (eg, weekly) meetings led by participant leaders who were an integral part of multidisciplinary teams might be helpful.^37^ A systematic review on the circumstances that lead to effective improvement collaboratives suggested that alignment of the improvement collaborative with existing supervisory structures, national systems and priorities, and leadership engagement might be key.^35^ Another review suggested that some aspects of teamwork and participation in specific improvement collaborative activities enhanced short-term success; and if quality improvement teams remained intact and continued to gather data, chances of long-term success were higher.^36^

More broadly, our findings have implications for how previous HCPPR results should be interpreted. The previous main HCPPR analyses compared the effectiveness of more than 100 strategies but only used effect sizes measured at the last time point in each study.^16^ For strategies with an effect that tends to decrease over time (eg, training only), the main HCPPR results might be conservative because they do not reflect higher effect levels in the first months after implementation. By the same logic, HCPPR results might be overestimated for strategies with an effect that tends to increase over time (eg, group problem-solving alone).

Furthermore, our findings have important implications for the design of future improvement strategies and their evaluations. The HCPPR found a total of 101 different strategies to improve facility-based HCP practice outcomes expressed as percentages. It is striking that, due to small numbers of studies evaluating each strategy, we were only able to assess effectiveness time trends for 5 (5%) of these 101 strategies. Such time trend results, however, are essential for decision-makers, as a strategy’s programmatic impact depends on the area under the quality-over-time curve (as well as the number of patients seen). Moreover, if a strategy’s effect is likely to wane over time, then programmes need to plan for follow-up or co-implemented strategies to prevent deteriorating quality. Our analysis highlights the utility of studies with multiple post-implementation measurements over a follow-up period that matches a timeframe that programmes require for improvements to be sustained (eg, at least 12 months). Programmes could use routinely collected data to evaluate strategies (if adequate data quality), as this could reduce the time and cost of evaluations. Furthermore, future research studies should use more standardised methods and attempt to replicate the findings for strategies with large, sustained effects that have weak supporting evidence. Finally, as the effectiveness of strategies is likely to vary in different contexts, regardless of the strategy that a programme chooses to implement, programmes need a long-term plan for monitoring their strategy’s effectiveness. Funders can help by strengthening systems that monitor quality and, to fill critical evidence gaps, supporting rigorous evaluations.

Our study had several limitations. Many included studies had a high risk of bias,^16^ and thus the associations between time and strategy effectiveness were supported by low-quality evidence. Bias due to secular trends was reduced by requiring all studies to have controls—either a separate control group, or for single-arm ITS studies, the baseline trend acted as a ‘historical’ control. Substantial contextual and methodological heterogeneity existed across the studies (eg, varying measurement methods, strategy definitions and strength of implementation). Most studies had short follow-up times and few follow-up measurements, which reduced our ability to evaluate time trends. Our analytical approach was intentionally designed to identify broad time trends across all studies in a strategy group, so results do not reflect differences within strategy groups. For instance, all training strategies were considered equivalent—although group in-service training and academic detailing are, from an implementation perspective, quite different. Furthermore, several strategy subgroups in our analysis included small numbers of studies, which prevented a full examination of confounding and limit the generalisability of the results. Also, we were unable to account for the possibility that, due to HCP reassignments that often occur over time, the HCPs exposed to a strategy at the beginning of a study’s follow-up period were, perhaps unintentionally, replaced by HCPs who did not receive the strategy and thus had lower performance. This phenomenon could help explain decreasing effectiveness trends. Additionally, our analyses were exploratory; thus, the associations between time and strategy effectiveness are not causal and should be interpreted in light of the limitations stated above. Finally, for ongoing strategies, we considered the post-implementation period to start after the first round of activities had been completed, which might have been before the strategies had their full impact. Readers who feel that several rounds of a strategy would have been needed before it had an effect could evaluate the trend starting several months into the post-implementation period.

The five strategies in this study focus on changing HCPs’ behaviours at the point of care. Although these strategies can be motivational and promote local commitment to quality, HCPs may revert to entrenched ways of doing things once training or supervision strategies are discontinued. One likely cause is that surrounding systems do not support quality improvement.^1 19^ In our studies, baseline HCP performance was low, with an average of only 37% across all practice outcomes. Low baseline performance combined with strategies that have relatively small effect sizes and an impact that tends to deteriorate over time mean that we need innovation and research to understand how to affect sustainable change on a larger scale in LMICs. Promising approaches have been articulated by expert groups, such as the macro-level structural reforms presented in the Lancet Global Health Commission on High Quality Health Systems: governing for quality, redesigning service delivery, reforming preservice education and igniting demand for quality in communities.^1^

## Conclusions

In conclusion, we found strategies with effects that, over time, tended to decline (training alone), remain unchanged (group problem-solving plus training) or increase (group problem-solving alone). Programmes relying solely on in-service training might need periodical refresher training or, better still, consider combining training with group problem-solving. Although more high-quality research is needed, our results are important for decision-makers as they choose strategies to improve HCP practices in LMICs, and they underscore the importance of monitoring strategy effectiveness by programmes. Regarding future research, our findings demonstrate the utility of studies with multiple post-implementation measurements over a programmatically relevant follow-up period so time trends of strategy effectiveness can be assessed.

## Data Availability

Data are available in a public, open access repository. The STATA code, data and an associated data dictionary are publicly available and can be found at: https://github.com/catherine-arsenault/Do-files-change-in-effectiveness-HCPPR-2020. Other data issued from the Health Care Provider Performance Review (HCPPR) can be found at: http://www.HCPperformancereview.org.
