# Supplementary material for: How does the effectiveness of strategies to improve healthcare provider practices in low-income and middle-income countries change after implementation? Secondary analysis of a systematic review
Source: BMJ Qual Saf. 2021 May 18;31(2):123–33. doi: 10.1136/bmjqs-2020-011717 (PMC8784997; doi:10.1136/bmjqs-2020-011717)

Supplementary materials for:

How does the effectiveness of strategies to improve health care provider practices in low- and middle-income countries change after implementation? Secondary analysis of a systematic review

Box A. Specifications for the statistical models

We used two-level random-intercept linear regression models with time-specific effect sizes clustered within studies and standard error estimation accounted for clustering at the study level. Because effect sizes tend to be lower when baseline performance is high (because there is less room for improvement), we also adjusted the models for baseline performance. All random-intercept linear regression modeling was conducted using the *mixed* command in STATA version 16.0. The models used a *multiple of the identity* structure of the covariance matrix for the random effects, which signifies that all variances are equal and all covariances are 0. The models were fit using maximum likelihood estimation. For statistical hypothesis testing, we set the type I error rate to α = 0.05.

Model equation:

$$\textit{Effect size}_{ij} = \beta_0 + \beta_1 \textit{time}_{ij} + \beta_2 \textit{baseline}_{ij} + u_i + \varepsilon_{ij}$$

Where $\textit{Effect size}_{ij}$ is the effect size for study *i* at follow-up time *j*
β_0 is the part of intercept common to all studies
β_1 is the regression coefficient for follow-up time
β_2 is the regression coefficient for the baseline effect size
u_i is the part of intercept that is specific to each study (the *random effect*)
ε_{ij} are the errors of the model

Model assumptions:

ε_{ij} are the errors of the model (the residuals) and are all assumed to be independent of each other and:

$$\varepsilon_{ij} \sim N(0, \sigma^2_{\varepsilon})$$

u_i is the random effect for study *i* and all u_i's are assumed to be independent of each other and:

$$u_i \sim N(0, \sigma^2_u)$$

ε_{ij} and u_i are all assumed to be independent of each other

The STATA do files used for the analyses and diagnostics plots for all regression models are available in a GitHub repository at: https://github.com/catherine-arsenault/Do-files-change-in-effectiveness-HCPPR-2020

Arsenault C, *et al*. *BMJ Qual Saf* 2022; 31:123–133. doi: 10.1136/bmjqs-2020-011717

Table A. Citations for included studies

Study id	Citation
100001	Angunawela II, Diwan VK, Tomson G. Experimental evaluation of the effects of drug information on antibiotic prescribing: a study in outpatient care in an area of Sri Lanka. International Journal of Epidemiology. 1991; 20(2):558-564.
400001	Loevinsohn BP, Guerrero ET, Gregorio SP. Improving primary health care through systematic supervision: a controlled field trial. Health Policy and Planning. 1995; 10(2):144-153.
500001	Santoso B, Suryawati S, Prawaitasari JE. Small group intervention vs. formal seminar for improving appropriate use. Social Science and Medicine, 1996; 42(8):1163-1168.
1000001	Bexell A, Lwando E, von Hofsten B, Tembo S, Eriksson B, Diwan VK. Improving drug use through continuing education: a randomized controlled trial in Zambia. J Clin Epidemiol, 1996; 49(3): 355-357.
2000001	Agyepong IA, Ansah E, Gyapong M, Adjei S, Barnish G, Evans D. Strategies to improve adherence to recommended chloroquine treatment regimes: a quasi-experiment in the context of integrated primary health care delivery in Ghana. Social Science and Medicine, 2002; 55:2215-2226.
3500001	Chowdhury AKA, Khan MOF, Matin A, Haque Z. Impact of standard treatment guidelines and small group training on prescribing for diarrhoea in under-five children in Bangladesh. INRUD News, 1995;5(2):20.
	Chowdhury AKA, Khan OF, Matin MA, Begum K, Galib MA. Effect of standard treatment guidelines with or without prescription audit on prescribing for acute respiratory tract infection (ARI) and diarrhoea in some thana health complexes (THCs) of Bangladesh. Bangladesh Medical Research Council Bulletin, 2007;33(1):21-30.
4200001	Flores R, Robles J, Burkhalter BR. Implementation and Evaluation of a Distance Education Course on the Management of Cholera and Diarrheal Diseases. Published for INCAP, PAHO/WHO, and USAID by the BASICS Project, Arlington, VA, 1998. Available online: http://www.basics.org/publications/pubs/implementation/contents.htm
	Flores R, Robles J, Burkhalter BR. Distance education with tutoring improves diarrhea case management in Guatemala. International Journal for Quality in Health Care, 2002; 14(supplement 1): 47-56.
4500001	Gonzalez Ochoa E, Armas Perez L, Bravo Gonzalez JR, Cabrales Escobar J, Rosales Corrales R, Abreu Suarez G. Prescription of antibiotics for mild acute respiratory infections in children. Bulletin of the Pan American Health Organization, 1996; 30(2): 106-117.
5200001	Hermida J, Robalino ME. Increasing compliance with maternal and child care quality standards in Ecuador. International Journal for Quality in Health Care, 2002; 14(supplement 1):25-34.
5900001	Kafle KK, Shrestha AD, Karkee SB, Yadav BP, Prasad RR, Shrestha N, Das PL, Pradhan YMS, Jha TN, Jha SS, Bhatta BR. Intervention test of training and supervision on dispensing practices. Report submitted to USAID/RPM/JSI Nepal. Kathmandu, Nepal. August 1995.
	Kafle KK, Shrestha N, Karkee SB, Prasad RR, Bhuju GB, Das PL. Intervention studies on rational use of drugs in public and private sector in Nepal. Nepal Medical College Journal. 2005; 7(1): 47-50.
6000001	Kafle KK, Shrestha AD, Karkee SB, Yadav BP, Prasad RR, Shrestha N, Das PL, Pradhan YMS, Jha TN, Jha SS, Bhatta BR. Intervention test of training and supervision on prescribing practices. Report submitted to USAID/RPM/JSI Nepal. Kathmandu, Nepal. August 1995.
	Kafle KK, Pradhan YMS, Shrestha AD, Karkee SB, Das PL, Shrestha N, Prasad RR. Better primary health care delivery through strengthening the existing supervision/monitoring. Poster presented at the International Conference on Improving Use of Medicines, Chiang Mai, Thailand, 1997.
	Kafle KK, Shrestha N, Karkee SB, Prasad RR, Bhuju GB, Das PL. Intervention studies on rational use of drugs in public and private sector in Nepal. Nepal Medical College Journal. 2005; 7(1): 47-50.

Study id	Citation
6100001	Kafle KK, Karkee SB, Shrestha N, Prasad RR, Bhuju GB, Das PL, Shrestha AD, Ross-Degnan D. Improving private drug sellers' practices for managing common health problems in Nepal. Journal of Nepal Health Research Council. 2013; 11(24):198-204.
	Kafle KK, Shrestha AD, Karkee SB, Das PL, Shrestha N, Prasad RR, Pradhan YMS, Quick JD, Ross-Degnan D, Shrestha BR, Baniya R, Adhikary R, Singh KK, Bhandari R, Lamichhane R, Lamichhane A, Upadhyaya S. Impact of action-oriented training and/or mailed print material on retailer practices: safe dispensing, correct advice, and appropriate referral for diarrhoea, ARI, and pregnancy. Unpublished report, Kathmandu, Nepal, 21 July 1998.
	Kafle KK, Shrestha N, Karkee SB, Prasad RR, Bhuju GB, Das PL. Intervention studies on rational use of drugs in public and private sector in Nepal. Nepal Medical College Journal. 2005; 7(1): 47-50.
6300001	Kafuko JM, Zirabamuzaale C, Bagenda D. Rational drug use in rural health units of Uganda: effect of national standard treatment guidelines on rational drug use. International Conferences on Improving Use of Medicines, Chiang Mai, Thailand, 1997. (accessed on July 20, 2006: http://mednet3.who.int/icium/icium1997/posters/2f3_text.html).
9190101	Bailey PE, Szaszdi JA, Schieber B. Analysis of the Vital Events Reporting System of the Maternal and Neonatal Health Project, Quetzaltenango, Guatemala. Technical Working Paper 3. December 1994. Working Paper prepared for USAID, Contract No. DPE 5966-Z-00-8083-00. available at: http://pdf.usaid.gov/pdf_docs/PNACE151.pdf, accessed Jan 14, 2009.
	MotherCare. Quetzaltenango Maternal and Neonatal Health Project. Pages 33-38. In: MotherCare, 1989-1993: country project descriptions. John Snow, Inc., USAID. September 1993. USAID Award/Agreement Number: DPE-5966-Z-00-8083-00. 57 pages.
	O'Rourke K. The effect of hospital staff training on management of obstetrical patients referred by traditional birth attendants. In: MotherCare Matters Newsletter and literature review on maternal and neonatal health and nutrition, 1996;5(4):4-7.
	Scheiber BA, Mejia M, Koritz S, Gonsalez C, Kwast BE. Medical audit of early neonatal deaths -- INCAP/Quetzaltenango Maternal and Neonatal Health Project. In: MotherCare Matters Newsletter and literature review on maternal and neonatal health and nutrition, 1996;5(4):7-12.
	Bailey PE, Szaszdi JA, Schieber B. Analysis of the Vital Events Reporting System of the Maternal and Neonatal Health Project, Quetzaltenango, Guatemala. In: MotherCare Matters Newsletter and literature review on maternal and neonatal health and nutrition, 1996;5(4):13-22.
	O'Rourke, K. The effect of hospital staff training on management of obstetrical patients referred by traditional birth attendants. International Journal of Gynecology & Obstetrics, 1995;48(Suppl):S95-S102.
	Bailey PE, Szaszdi JA, Glover L. Obstetric complications: does training traditional birth attendants make a difference? Pan American Journal of Public Health, 2002;11(1):15-23.
	O'Rourke K. Evaluación de un programa de capacitación de parteras tradicionales en Quetzaltenango, Guatemala. Boletín de la Oficina Sanitaria Panamericana, 1995; 119 (6): 503 - 514.
	Kwast BE, Koblinsky MA. Starting maternity care programmes in developing countries to reduce maternal mortality. Contemporary Reviews in Obstetrics & Gynaecology, 1995;7(4):220-225.
	Kwast BE. Building a community-based maternity program. International Journal of Gynecology & Obstetrics, 1995;48 Suppl:S67-82.
	Kwast BE. Reduction of maternal and perinatal mortality in rural and peri-urban settings: what works? European Journal of Obstetrics & Gynecology and Reproductive Biology, 1996;69(1):47-53.
9600001	Podhipak A, Varavithya W, Punyaratabandhu P, Vathanophas K, Sangchai R. Impact of an educational program on the treatment practices of diarrheal diseases among pharmacists and drugsellers. Southeast Asian Journal of Tropical Medicine and Public Health, 1993;24(1):32-39.
10300001	Ruangkanchanasetr, S. Laboratory investigation utilization in pediatric out-patient department Ramathibodi Hospital. J Med Assoc Thai, 1993; 76(Suppl 2): 194-208.

Study id	Citation
10300002	Ruangkanchanasetr, S. Laboratory investigation utilization in pediatric out-patient department Ramathibodi Hospital. J Med Assoc Thai, 1993; 76(Suppl 2): 194-208.
11400001	Thuo HM, Gelders SFAM. Multi-feedback approach to rational drug use: using inpatient drug use indicators as an intervention tool. Presented at International Conference on the Rational Use of Medicines (ICIUM), 1997.
12000001	Widyastuti S, Dwiprahasto I, Andajaningsih, Bakri Z. The impact of problem-based rational drug use training on prescribing practices, cost reallocations and savings in primary care facilities. Poster presented at International Conferences on Improving Use of Medicines (ICIUM), Chiang Mai, Thailand, 1997. Available on the web at: http://mednet3.who.int/icium/icium1997/posters/2b4_text.html, accessed 20 July 2006.
16100001	Mohagheghi MA, Mosavi-Jarrahi A, Khatemi-Moghaddam M, Afhami A, Khodi S, Azemoodeh O. Community-based outpatient practice of antibiotics use in Tehran. Pharmacoepidemiology and Drug Safety, 2005; 14: 135-38.
17190101	Trap B, Todd CH, Moore H, Laing R. The impact of supervision on stock management and adherence to treatment guidelines: a randomized controlled trial. Health Policy and Planning, 2001;16(3):273-280.
27600001	Winch PJ, Bagayoko A, Diawara A, Kané M, Thiéro F, Gilroy K, Daou Z, Berthé Z, Swedberg E. Increases in correct administration of chloroquine in the home and referral of sick children to health facilities through a community-based intervention in Bougouni District, Mali. Transactions of the Royal Society of Tropical Medicine & Hygiene, 2003;97(5):481-490.
	Gilroy K, Winch PJ, Diawara A, Swedburg E, Thiéro F, Kané M, Daou Z, Berthé Z, Bagayoko A. Impact of IMCI training and language used by provider on quality of counseling provided to parents of sick children in Bougouni District, Mali. Patient Education and Counseling, 2004;54:35-44.
28600001	Baral JP, Bhattarai S, Thapa MR, Ghimire K, Burathoki K. Effectiveness of training of basic health workers in leprosy control programme. Indian Journal of Leprosy, 1998; 70(suppl): 23S-31S.
28700001	Bawa SB, Olumide EAA. The effect of training on the reporting of notifiable diseases among health workers in Yobe State, Nigeria. The Nigerian Postgraduate Medical Journal, 2005; 12(1):1-5.
	Bawa SB, Olumide EAA, Umar US. The knowledge, attitude and practices of the reporting of notifiable diseases among health workers in Yobe State, Nigeria. African Journal of Medicine and Medical Sciences, 2003; 32(1):49-53.
30600001	Kounnavong S, Wahlstrom R, Sisounthone B, Panyanouvong A, Southammavong T. Effectiveness of feedback for improving case management of malaria, diarrhea, and pneumonia: a randomized controlled trial at provincial hospitals in Lao PDR. Paper presented at: Second International Conference on Improving Use of Medicines (ICIUM); March 30 - April 2, 2004; Chiang Mai, Thailand. [cited April 11, 2010]. Available from: http://www.icium.org/icium2004/resources/ppt/O_MA007.ppt.
	Wahlstrom R, Kounnavong S, Sisounthone B, Phanyanouvong A, Southammavong T, Eriksson B, Tomson G. Effectiveness of feedback for improving case management of malaria, diarrhoea and pneumonia-a randomized controlled trial at provincial hospitals in Lao PDR. Tropical Medicine and International Health, 2003;8(10):901-909.
33600001	Arreola-Risa C, Mock C, Herrera-Escamilla AJ, Contreras I, Vargas J. Cost-effectiveness and benefit of alternatives to improve training for prehospital trauma care in Mexico. Prehospital and Disaster Medicine, 2004;19(4):318-325.
	Arreola-Risa C, Mock CN, Lojero-Wheatly L, de la Cruz O, Garcia C, Canavati-Ayub F, Jurkovich GJ. Low-cost Improvements in Prehospital Trauma Care in a Latin American City. The Journal of Trauma: Injury, Infection, and Critical Care, 2000; 48(1): 119-124.
39300001	Huang J, Jiang D, Wang X, Liu Y, Fennie K, Burgess J, Williams AB. Changing knowledge, behavior, and practice related to universal precautions among hospital nurses in China. The Journal of Continuing Education in Nursing, 2002; 33(5):217-224.
47600001	Ratanajamit C, Chongsuvivatwong V, Geater AF. A randomized controlled educational intervention on emergency contraception among drugstore personnel in southern Thailand. J Am Med Womens Assoc. 2002; 57(4): 196-9, 207.
48400001	Ramarao S, Mir AM. Transforming relationships in Pakistani villages. Quality / Calidad / Qualité. 2004; 15: 20-34.

Study id	Citation
	Sathar Z, Jain A, Ramarao S, ul Haque M, Kim J. Introducing client-centered reproductive health services in a Pakistani setting. Studies in Family Planning. 2005; 36(3): 221-234.
53400001	Uys LR, Minnaar A, Simpson B, Reid S. The effect of two models of supervision on selected outcomes. Journal of Nursing Scholarship. 2005;37(3):282-8.
62100001	Pagaiya N, Garner P. Primary care nurses using guidelines in Thailand: a randomized controlled trial. Tropical Medicine and International Health, 2005;10(5):471-477.
63100001	Faxelid E, Ahlberg M, Freudenthal S, Ndulo J, Krantz I. Quality of STD care in Zambia. Impact of training in STD management. International Journal for Quality in Health Care. 1997; 9(5): 361-366.
67100001	Rea MF, Venancio SI, Martines JC, Savage F. Counselling on breastfeeding: assessing knowledge and skills. Bulletin of the World Health Organization, 1999; 77(6): 492-498.
67200001	Meyer JC, Summers RS, Moller H. Randomized, controlled trial of prescribing training in a South African province. Medical Education, 2001(9); 35: 833-840.
68390101	Costello M, Lacuesta M, RamaRao S, Jain A. A client-centered approach to family planning: the Davao Project. Studies in Family Planning. 2001; 32(4):302-14.
	Jain A, RamaRao S, Kim J, Costello M. Evaluation of an intervention to improve quality of care in family planning programme in the Philippines. Journal of Biosocial Science. 2012; 44(1):27-41.
68800001	Mukti AG, Treloar C, Suprawimbarti, Asdie AH, D'Este K, Higginbotham N, Heller R. A universal precautions education intervention for health workers in Sardjito and Pku Hospital, Indonesia. Southeast Asia Journal of Tropical Medicine and Public Health. 2000; 31(2): 405-411.
68900001	Lueveswanij S, Nittayananta W, Robison VA. Changing knowledge, attitudes, and practices of Thai oral health personnel with regard to AIDS: an evaluation of an educational intervention. Community Dental Health. 2000; 17:165-171.
69200001	Johnson KW, Young LC, Bryant D, Suresh G, Bettler RF (Pacific Institute for Research and Evaluation- Louisville Center), Berbaum ML (Health Research and Policy Centers, University of Illinois at Chicago). The Daytop drug-free treatment training evaluation. Research Monograph. Washington DC. Bureau for International Narcotics and Law Enforcement Affairs, Department of State. July 2000. 77p.
	Johnson KW, Young LC, Suresh G, Berbaum ML. Drug abuse treatment training in Peru. A social policy experiment. Evaluation Review. 2002; 26(5): 480-519.
105500001	Syhakhang, L. The quality of private pharmacy services in a province of Lao PDR: perceptions, practices and regulatory enforcements. Doctor's Thesis from Karolinska Institutet, Division of International Health (IHCAR), Department of Public Health Sciences, 2002; Available at: http://diss.kib.ki.se/2002/91-7349-357-0/thesis.pdf. Accessed January 29, 2010.
	Stenson B, Syhakhang L, Lundborg CS, Eriksson B, Tomson G. Private Pharmacy Practice and Regulation: A Randomized Trial in Lao P.D.R. International Journal of Technology Assessment in Health Care, 2001; 17(4): 579-589.
108100001	Wang H, Fennie K, He G, Burgess J, Williams AB. A training programme for prevention of occupational exposure to bloodborne pathogens: impact on knowledge, behaviour and incidence of needle stick injuries among student nurses in Changsha, People's Republic of China. Journal of Advanced Nursing, 2003; 41(2): 187-194.
120700001	Tavrow P, Malianga L, Kariuki M. Using problem-solving teams to improve compliance with IMCI guidelines in Kenya. Bethesda (MD): Quality Assurance Project (US); 2004. 13 p. Operations Research Report. Published for the U.S. Agency for International Development (USAID).
124100001	Lin YS, Hermida J, Hernandez F, Nunez O, Urbina L. Using quality assessment to improve maternal care in Nicaragua. Quality Assurance Project Case Study. Bethesda (MD): Quality Assurance Project (US); 2003 May. 23 p. Published for U.S. Agency for International Development. Contract No: HRN-C-00-96-90013.

Study id	Citation
132600001	Kafle KK, Shrestha AD, Shrestha N, Prasad RR, Das PL, Bhuju GB. Test of Strategies for Implementing STS in Improving Use of Drugs. INRUD, Nepal; New Baneswore, Kathmandu, Nepal; March, 2001.
	Kafle KK, Shrestha AD, Shrestha N, Prasad RR, Das PL, Bhuju GB. Test of sustainability of strategy in implementing STS for improving use of drugs. Technical Report, INRUD, Nepal, 2002. available at: http://www.inrud-nepal.org.np/publications/STS-Test%20of%20sustainability.pdf, accessed Dec 30, 2009.
	Kafle K, Karkee SB, Prasad RR, Bhuju GB, Shrestha N, Das PL, Shrestha AD. Evaluation of a successful intervention for sustainability and effects in post research phase. Kathmandu University Medical Journal, 2006; 4(1): 61-64.
	Kafle KK, Shrestha N, Karkee SB, Prasad RR, Bhuju GB, Das PL. Intervention studies on rational use of drugs in public and private sector in Nepal. Nepal Medical College Journal, 2005; 7(1): 47-50.
133200001	Akici A, Kalaca S, Ugurlu MU, Karaalp A, Cali S, Oktay S. Impact of a short postgraduate course in rational pharmacotherapy for general practitioners. British Journal of Clinical Pharmacology, 2004;57(3): 310-321.
133300001	Eltayeb IB, Awad AI, Mohamed-Salih MS, Daffa-Alla MA, Ahmed MB, Ogail MA, Matowe L. Changing the prescribing patterns of sexually transmitted infections in the White Nile Region of Sudan. Sexually Transmitted Infections, 2005; 81(5):426-427.
133400001	Shrestha N, Samir KC, Baltussen R, Kafle KK, Bishai D, Niessen L. Practical approach to lung health in Nepal: better prescribing and reduction of cost. Tropical Medicine and International Health, 2006;11(5):765-772.
134500001	Kotwani A, Gupta U, Suri JC, Chaudhury RR. Impact of educational intervention on prescribing behavior and cost of therapy in bronchial asthma in colony hospitals of Delhi. Poster presentation at 2nd ICIUM conference, Chiang Mai, Thailand, Mar 30-Apr 2, 2004. author email: anitakotwani@msn.com, available at: http://archives.who.int/icium/icium2004/resources/ppt/AD035.ppt, accessed Sept 11, 2008.
	Chaudhury RR, Parameswar R, Gupta U, Sharma S, Tekur U, Bapna JS. Quality medicines for the poor: experience of the Delhi programme on rational use of drugs. Health Policy and Planning, 2005 Mar; 20(2):124-36.
134800001	Dehli Society for Promotion of Rational Use of Drugs, INDIA-WHO Essential Drugs Programme. Research on Rational Drug Use in India: A Glimpse. 2001.
	Euro Health Group Consultants. Final Report. Delhi Society for the Promotion of Rational Use of Drugs (DSPRUD). External evaluation of the activities in the past 5 years, New Delhi, 7-24 October 2002. January 2003.
	Sharma S, Gupta U, Chaudhury RR, Bapna JS. Prescribing behaviour of physicians. Journal of Health Management, 2002; 4(1): 55-71.
134900001	Awad AI, Eltayeb IB, Baraka OZ. Changing antibiotics prescribing practices in health centers of Khartoum State, Sudan. European Journal of Clinical Pharmacology, 2006; 62(2):135-142.
135000001	Teng CL, Achike FI, Phua KL, Nurjahan MI, Mastura I, Asiah HN, Mariam AM, Narayanan S, Norsiah A, Sabariah I, Shariah I, Rokiah KS, Zubaidah MA, Koh CN, Rosnah I. Modifying antibiotic prescribing: the effectiveness of academic detailing plus information leaflet in a Malaysian primary care setting. Medical Journal of Malaysia. 2006; 61(3): 323-331.
135400001	Qidwai W, Krishanani MK, Hashmi S, Afridi M, Ali RA. Private drug seller's education in improving prescribing practices: Results of an interventional study from Rural Singh, Pakistan. 16 p.
	Qidwai W, Krishanani MK, Hashmi S, Afridi M, Ali RA. Private drug sellers' education in improving prescribing practices. Journal of the College of Physicians and Surgeons--Pakistan. 2006;16(12):743-746.
138600001	Levav I, Kohn R, Montoya I, Palacio C, Rozic P, Solano I, Valentini W, Vicente B, Morales JC, Eigueta FE, Saravanan Y, Miranda CT, Sartorius N. Training Latin American primary care physicians in the WPA module on depression: results of a multicenter trial. Psychological Medicine. 2005;35(1):35-45.

Study id	Citation
	Valentini W, Levav I, Kohn R, Miranda CT, Mello AA, Mello MF, Ramos CP. Treinamento de clínicos para o diagnóstico e tratamento da depressão [An educational training program for physicians for diagnosis and treatment of depression]. Revista de saúde pública. 2004;38(4):522-8.
	Vicente B, Kohn R, Levav I, Espejo F, Saldivia S, Sartorius N. Training primary care physicians in Chile in the diagnosis and treatment of depression. Journal of Affective Disorders. 2007;98(1-2):121-7.
144200001	Odusanya OO, Oyediran MA. The effect of an educational intervention on improving rational drug use. Nigerian Postgraduate Medical Journal. 2004 Jun;11(2):126-131.
149400001	Isah AO, Isah EC, Okojie OH, Ohaju-Obodo JO. An interventional study to promote rational use of drugs with a newly developed prescribing indicator reference standards in Nigeria. Department of Medicine and Community Health, University of Benin/Teaching Hospital. 1999.
	Isah AO. Can drug use indicators serve as interventional tools in promoting rational use of drugs? A field testing study in Nigeria. A Report to the Drug Action Programme - World Health Organization (DAP-WHO), DAP-WHO PROJECT GLOBAL/RUS/ISAH (E19/181/49), 1999.
149600001	Akoria OA, Isah AO. Prescription writing in publilc and private hospitals in Benin City, Nigeria: the effects of an educational intervention. Canadian Journal of Clinical Pharmacology. 2008; 15(2): e295-e305.
195400001	Chowdhury AKA, Khan OF, Matin MA, Begum K, Galib MA. Study of effect of standard treatment guidelines with or without audit on prescribing for diarrhoea and acute respiratory infection in some government health facilities in Bangladesh. Unpublished manuscript.
	Chowdhury AKA, Khan OF, Matin MA, Begum K, Galib MA. Effect of standard treatment guidelines with or without prescription audit on prescribing for acute respiratory tract infection (ARI) and diarrhoea in some Thana Health Complexes (THCs) of Bangladesh. Bangladesh Medical Research Council Bulletin, 2007; 33(1):21-30.
197400001	Ngasala B, Mubi M, Warsame M, Petzold MG, Massele AY, Gustafsson LL, Tomson G, Premji Z, Bjorkman A. Impact of training in clinical and microscopy diagnosis of childhood malaria on antimalarial drug prescription and health outcome at primary health care level in Tanzania: a randomized controlled trial. Malaria Journal. 2008; 7:199.
201000001	Senarath U, Fernando D, Rodrigo I. Effect of training for care providers on practice of essential newborn care in hospitals in Sri Lanka. Journal of Obstetric Gynecologic and Neonatal Nursing. 2007 Nov-Dec;36(6):531-541.
205900001	Rowe AK, Osterholt DM, Kouamé J, Piercefield E, Herman KM, Onikpo F, Lama M, Deming MS. Trends in health worker performance after implementing the Integrated Management of Childhood Illness strategy in Benin. Tropical Medicine and International Health. 2012;17(4):438-46.
	Osterholt DM, Onikpo F, Lama M, Deming MS, Rowe AK. Improving pneumonia case-management in Benin: a randomized trial of a multi-faceted intervention to support health worker adherence to Integrated Management of Childhood Illness guidelines. Human Resources for Health. 2009;7 (77): 1-13.
	Rowe AK, Onikpo F, Lama M, Osterholt DM, Rowe SY, Deming MS. A multifaceted intervention to improve health worker adherence to integrated management of childhood illness guidelines in Benin. American Journal of Public Health. 2009; 99(5): 837-846.
	Rowe AK, Onikpo F, Lama M, Deming MS. The rise and fall of supervision in a project designed to strengthen supervision of Integrated Management of Childhood Illness in Benin. Health Policy and Planning. 2010; 25(2): 125-134.
231300001	León FR, Brambila C, de la Cruz M, Bratt J, García Colindres J, Vásquez B, Morales C. Testing balanced counseling to improve provider-client interaction in Guatemala's MOH clinics. Population Council, Washington, DC. 2003 October. 36p. Basic funding provided by the US Agency for International Development. FRONTIERS Cooperative Agreement No.: HRN-A-00-98-00012-00. Project No.: 5801 13052.
	León FR, Brambila C, de la Cruz M, García Colindres J, Morales C, Vásquez B. Providers' compliance with the balanced counseling strategy in Guatemala. Studies in Family Planning. 2005; 36(2): 117-126.

Study id	Citation
234200001	Catsambas TT, Franco LM, Gutmann M, Knebel E, Hill P, Lin Y-S. Evaluating health care collaboratives: the experience of the Quality Assurance Project. Bethesda (MD): University Research Co., LLC (URC); 2008 June. 89 p. Collaborative Evaluation Series. Published by the USAID Health Care Improvement Project.
	Ethier K. Developing evidence-based standards for pregnancy-induced hypertension in Russia. Quality Assurance Project Case Study. Bethesda (MD): Published for the U.S. Agency for International Development (USAID) by the Quality Assurance Project. 2001; 24 p.
	Abdallah H, Chernobrovkina O, Korotkova A, Massoud R, Burkhalter B. Improving the quality of care for women with pregnancy-induced hypertension reduces costs in Tver, Russia. Operations Research Results 2(4). Bethesda (MD): Published for the United States Agency for International Development (USAID) by the Quality Assurance Project. 2002; 16 p.
	Massoud MR. Applying modern quality improvement methodology to maternal and child health in Tver Oblast, Russian Federation. QA Brief. 2001; 9(2):28-32.
	USAID Health Care Improvement Project. The Improvement Collaborative: an approach to rapidly improve health care and scale up quality services. Bethesda (MD): University Research Co., LLC (URC); 2008 June. 20 p. Published by the USAID Health Care Improvement Project.
	Franco LM, Marquez L. Effectiveness of collaborative improvement: evidence from 27 applications in 12 less-developed and middle-income countries. British Medical Journal Quality & Safety. 2011; 20(8):658-65.
235900001	Westphal MF, Taddei JA, Venancio SI, Bogus CM. Breast-feeding training for health professionals and resultant institutional changes. Bulletin of the World Health Organization. 1995; 73(4): 461-468.
237000001	Hizaamu RNB, Waluga P. Total quality management. PowerPoint presentation presented at the Jinja District Local Government Health Management Team Quarterly Feedback Meeting; 2005 October; Uganda, and the HIV & AIDS Fellowship Programme Annual Dissemination Workshop; 2005 December 2; Uganda.
243800001	Yudatiningsih I (Sleman District Health Office), Sunartono H (Sleman District Health Office), Suryawati S (Center for Clinical Pharmacology and Drug Policy Studies, Gadjah Mada University, Yogyakarta, Indonesia). The importance of feedback to enhance the impact of effective interventions to reduce antibiotics in acute respiratory-tract infection. Presented at: The Second International Conference on Improving Use of Medicines; 2004 March 30 - April 2; Chiang Mai, Thailand. Available from: http://www.icium.org/icium2004/resources/ppt/AM010.ppt, accessed April 9, 2010.
243900001	Yudatiningsih I (Sleman District Health Office, Indonesia), Sunartono H (Sleman District Health Office, Indonesia). Long-term impact of an MTP approach to reduce inappropriate use of antibiotics in acute respiratory-tract infection. Presented at The Second International Conference on Improving Use of Medicines; 2004 March 30 - April 2; Chiang Mai, Thailand. Available from: http://www.icium.org/icium2004/resources/ppt/AM018.ppt, accessed April 9, 2010.
247600001	Ofei F, Forson A, Tetteh R, Ofori-Adjei D. An intervention to improve antibiotic prescribing habits of doctors in a teaching hospital. Presented at: Second International Conference on Improving Use of Medicines (ICIUM); March 30 - April 2, 2004; Chiang Mai, Thailand [cited April 10, 2010]. 13 p. Available from: http://www.icium.org/icium2004/resources/ppt/AD029.ppt
255500001	Franco LM, Marquez L. Effectiveness of collaborative improvement: evidence from 27 applications in 12 less-developed and middle-income countries. British Medical Journal Quality & Safety. 2011; 20(8):658-65.
	Catsambas TT, Franco LM, Gutmann M, Knebel E, Hill P, Lin Y-S. Evaluating health care collaboratives: the experience of the Quality Assurance Project. Bethesda (MD): University Research Co., LLC (URC); 2008 June. 89 p. Collaborative Evaluation Series. Published by the USAID Health Care Improvement Project.
	Franco LM, Marquez L, Ethier K, Balsara Z, Isenhower W. Results of collaborative improvement: effects on health outcomes and compliance with evidence-based standards in 27 applications in 12 countries. Bethesda (MD): University Research Co., LLC (URC); 2009 December. 48 p. Collaborative Evaluation Series. Published by the USAID Health Care Improvement Project.

Study id	Citation
	USAID Health Care Improvement Project. The Improvement Collaborative: an approach to rapidly improve health care and scale up quality services. Bethesda (MD): University Research Co., LLC (URC); 2008 June. 20 p. Published by the USAID Health Care Improvement Project.
	Broughton E, Saley Z, Boucar M, Alagane D, Hill K, Marafa A, Asma Y, Sani K. Cost-effectiveness of a quality improvement collaborative for obstetric and newborn care in Niger. International Journal of Health Care Quality Assurance. 2013;26(3):250-61.
	Franco LM, Webb L. Niger Site Visit Report. Unpublished report prepared for the U.S. Agency for International Development (USAID) by the USAID Health Care Improvement Project and the Quality Assurance Project. 2008 February. pp iii-v and 30-42.
255500002	Franco LM, Marquez L. Effectiveness of collaborative improvement: evidence from 27 applications in 12 less-developed and middle-income countries. British Medical Journal Quality & Safety. 2011; 20(8):658-65.
	Catsambas TT, Franco LM, Gutmann M, Knebel E, Hill P, Lin Y-S. Evaluating health care collaboratives: the experience of the Quality Assurance Project. Bethesda (MD): University Research Co., LLC (URC); 2008 June. 89 p. Collaborative Evaluation Series. Published by the USAID Health Care Improvement Project.
	Franco LM, Marquez L, Ethier K, Balsara Z, Isenhower W. Results of collaborative improvement: effects on health outcomes and compliance with evidence-based standards in 27 applications in 12 countries. Bethesda (MD): University Research Co., LLC (URC); 2009 December. 48 p. Collaborative Evaluation Series. Published by the USAID Health Care Improvement Project.
	USAID Health Care Improvement Project. The Improvement Collaborative: an approach to rapidly improve health care and scale up quality services. Bethesda (MD): University Research Co., LLC (URC); 2008 June. 20 p. Published by the USAID Health Care Improvement Project.
	Broughton E, Saley Z, Boucar M, Alagane D, Hill K, Marafa A, Asma Y, Sani K. Cost-effectiveness of a quality improvement collaborative for obstetric and newborn care in Niger. International Journal of Health Care Quality Assurance. 2013;26(3):250-61.
	Franco LM, Webb L. Niger Site Visit Report. Unpublished report prepared for the U.S. Agency for International Development (USAID) by the USAID Health Care Improvement Project and the Quality Assurance Project. 2008 February. pp iii-v and 30-42.
255500003	Franco LM, Marquez L. Effectiveness of collaborative improvement: evidence from 27 applications in 12 less-developed and middle-income countries. British Medical Journal Quality & Safety. 2011; 20(8):658-65.
	Catsambas TT, Franco LM, Gutmann M, Knebel E, Hill P, Lin Y-S. Evaluating health care collaboratives: the experience of the Quality Assurance Project. Bethesda (MD): University Research Co., LLC (URC); 2008 June. 89 p. Collaborative Evaluation Series. Published by the USAID Health Care Improvement Project.
	Franco LM, Marquez L, Ethier K, Balsara Z, Isenhower W. Results of collaborative improvement: effects on health outcomes and compliance with evidence-based standards in 27 applications in 12 countries. Bethesda (MD): University Research Co., LLC (URC); 2009 December. 48 p. Collaborative Evaluation Series. Published by the USAID Health Care Improvement Project.
	USAID Health Care Improvement Project. The Improvement Collaborative: an approach to rapidly improve health care and scale up quality services. Bethesda (MD): University Research Co., LLC (URC); 2008 June. 20 p. Published by the USAID Health Care Improvement Project.
	Franco LM, Webb L. Niger Site Visit Report. Unpublished report prepared for the U.S. Agency for International Development (USAID) by the USAID Health Care Improvement Project and the Quality Assurance Project. 2008 February. pp iii-v and 30-42.
255500005	Catsambas TT, Franco LM, Gutmann M, Knebel E, Hill P, Lin Y-S. Evaluating health care collaboratives: the experience of the Quality Assurance Project. Bethesda (MD): University Research Co., LLC (URC); 2008 June. 89 p. Collaborative Evaluation Series. Published by the USAID Health Care Improvement Project.
	Ethier K. Developing evidence-based standards for pregnancy-induced hypertension in Russia. Quality Assurance Project Case Study. Bethesda (MD): Published for the U.S. Agency for International Development (USAID) by the Quality Assurance Project. 2001; 24 p.

Study id	Citation
	Abdallah H, Chernobrovkina O, Korotkova A, Massoud R, Burkhalter B. Improving the quality of care for women with pregnancy-induced hypertension reduces costs in Tver, Russia. Operations Research Results 2(4). Bethesda (MD): Published for the United States Agency for International Development (USAID) by the Quality Assurance Project. 2002; 16 p.
	Massoud MR. Applying modern quality improvement methodology to maternal and child health in Tver Oblast, Russian Federation. QA Brief. 2001; 9(2):28-32.
	USAID Health Care Improvement Project. The Improvement Collaborative: an approach to rapidly improve health care and scale up quality services. Bethesda (MD): University Research Co., LLC (URC); 2008 June. 20 p. Published by the USAID Health Care Improvement Project.
	Franco LM, Marquez L. Effectiveness of collaborative improvement: evidence from 27 applications in 12 less-developed and middle-income countries. British Medical Journal Quality & Safety. 2011; 20(8):658-65.
255500009	Catsambas TT, Franco LM, Gutmann M, Knebel E, Hill P, Lin Y-S. Evaluating health care collaboratives: the experience of the Quality Assurance Project. Bethesda (MD): University Research Co., LLC (URC); 2008 June. 89 p. Collaborative Evaluation Series. Published by the USAID Health Care Improvement Project.
	Franco LM, Marquez L, Ethier K, Balsara Z, Isenhower W. Results of collaborative improvement: effects on health outcomes and compliance with evidence-based standards in 27 applications in 12 countries. Bethesda (MD): University Research Co., LLC (URC); 2009 December. 48 p. Collaborative Evaluation Series. Published by the USAID Health Care Improvement Project.
	USAID Health Care Improvement Project. The Improvement Collaborative: an approach to rapidly improve health care and scale up quality services. Bethesda (MD): University Research Co., LLC (URC); 2008 June. 20 p. Published by the USAID Health Care Improvement Project.
	Franco LM, Marquez L. Effectiveness of collaborative improvement: evidence from 27 applications in 12 less-developed and middle-income countries. British Medical Journal Quality & Safety. 2011; 20(8):658-65.
255500010	Catsambas TT, Franco LM, Gutmann M, Knebel E, Hill P, Lin Y-S. Evaluating health care collaboratives: the experience of the Quality Assurance Project. Bethesda (MD): University Research Co., LLC (URC); 2008 June. 89 p. Collaborative Evaluation Series. Published by the USAID Health Care Improvement Project.
	Franco LM, Marquez L, Ethier K, Balsara Z, Isenhower W. Results of collaborative improvement: effects on health outcomes and compliance with evidence-based standards in 27 applications in 12 countries. Bethesda (MD): University Research Co., LLC (URC); 2009 December. 48 p. Collaborative Evaluation Series. Published by the USAID Health Care Improvement Project.
	USAID Health Care Improvement Project. The Improvement Collaborative: an approach to rapidly improve health care and scale up quality services. Bethesda (MD): University Research Co., LLC (URC); 2008 June. 20 p. Published by the USAID Health Care Improvement Project.
	Wittcoff A, Furth R, Nabwire J, Crigler L. Baseline assessment of HIV service provider productivity and efficiency in Uganda. Technical Report. 2010 Sep; University Research Co., LLC. Published for the US Agency for International Development by QAP.
	Franco LM, Marquez L. Effectiveness of collaborative improvement: evidence from 27 applications in 12 less-developed and middle-income countries. British Medical Journal Quality & Safety. 2011; 20(8):658-65.
255500012	Catsambas TT, Franco LM, Gutmann M, Knebel E, Hill P, Lin Y-S. Evaluating health care collaboratives: the experience of the Quality Assurance Project. Bethesda (MD): University Research Co., LLC (URC); 2008 June. 89 p. Collaborative Evaluation Series. Published by the USAID Health Care Improvement Project.
	Franco LM, Marquez L, Ethier K, Balsara Z, Isenhower W. Results of collaborative improvement: effects on health outcomes and compliance with evidence-based standards in 27 applications in 12 countries. Bethesda (MD): University Research Co., LLC (URC); 2009 December. 48 p. Collaborative Evaluation Series. Published by the USAID Health Care Improvement Project.

Study id	Citation
	USAID Health Care Improvement Project. The Improvement Collaborative: an approach to rapidly improve health care and scale up quality services. Bethesda (MD): University Research Co., LLC (URC); 2008 June. 20 p. Published by the USAID Health Care Improvement Project.
	Wittcoff A, Furth R, Nabwire J, Crigler L. Baseline assessment of HIV service provider productivity and efficiency in Uganda. Technical Report. 2010 Sep; University Research Co., LLC. Published for the US Agency for International Development by QAP.
	Franco LM, Marquez L. Effectiveness of collaborative improvement: evidence from 27 applications in 12 less-developed and middle-income countries. British Medical Journal Quality & Safety. 2011; 20(8):658-65.
257800001	Charandabi SMA, Vahidi R, Marions L, Wahlström R. Effect of a peer-educational intervention on provider knowledge and reported performance in family planning services: a cluster randomized trial. BMC Medical Education. 2010; 10:11.
257800002	Charandabi SMA, Vahidi R, Marions L, Wahlström R. Effect of a peer-educational intervention on provider knowledge and reported performance in family planning services: a cluster randomized trial. BMC Medical Education. 2010; 10:11.
258000001	Khanal S. Study the impact of academic detailing on childhood diarrhoea management to the primary healthcare providers in Nepal [master's thesis]. [Penang (Malaysia)]: Universiti Sains Malaysia; 2010, 217 p.
	Khanal S, Ibrahim MIM, Shankar PR, Palaian S, Mishra P. Evaluation of academic detailing programme on childhood diarrhoea management by primary healthcare providers in Banke district of Nepal. Journal of Health, Population and Nutrition. 2013;31(2):231-42.
	Khanal S, Palaian S, Shankar PR, Mishra P, Ibrahim MIM. Impact of educational outreach visits by pharmacists on treatment of childhood diarrhoea - initial findings from Banke district, Nepal. South East Asian Journal of Medical Education. 2009;3(2):76-77.
258100001	Bello DA, Hassan ZI, Afolaranmi TO, Tagurum YO, Chirdan OO, Zoakah AI. Supportive supervision: an effective intervention in achieving high quality malaria case management at primary health care level in Jos, Nigeria. Annals of African Medicine. 2013;12(4):243-51.
258900001	Baumgartner JN, Morroni C, Mlobeli RD, Otterness C, Buga G, Chen M. Impact of a provider job aid intervention on injectable contraceptive continuation in South Africa. Studies in Family Planning. 2012;43(4):305-14.
	Baumgartner JN, Morroni C, Mlobeli RD, Otterness C, Myer L, Janowitz B, Stanback J, Buga G. Timeliness of contraceptive reinjections in South Africa and its relation to unintentional discontinuation. International Family Planning Perspectives. 2007;33(2):66-74.
267400001	Fritz J, Walker D, Cohen S, Angeles G, Lamadrid-Figueroa H. Can a simulation-based training program impact the use of evidence based routine practices at birth? Results of a hospital-based controlled trial in Mexico. Poster session presented at: Global Maternal Newborn Health Conference; 2015 Oct 18-21; Mexico City, Mexico.
	Lamadrid-Figueroa H. Impact of PRONTO: results of a cluster randomized trial in Mexico. Paper presented at: Global Maternal Newborn Health Conference; 2015 Oct 18-21; Mexico City, Mexico.
	Fritz J, Walker D, Cohen S, Angeles G, Lamadrid-Figueroa H. Can a simulation-based training program impact the use of evidence based routine practices at birth? Results of a hospital-based controlled trial in Mexico. Currently under review. 2016.
	Walker D, Cohen S, Fritz J, Olvera M, Lamadrid-Figueroa H, Cowan JG, Hernandez DG, Dettinger JC, Fahey JO. Team training in obstetric and neonatal emergencies using highly realistic simulation in Mexico: impact on process indicators. BMC Pregnancy and Childbirth. 2014; 14:367.
	Walker D, Cohen SR, Fritz J, Olvera-Garcia M, Zelek ST, Fahey JO, Romero-Martinez M, Montoya-Rodriguez A, Lamadrid-Figueroa H. Impact evaluation of PRONTO Mexico: a simulation-based program in obstetric and neonatal emergencies and team training. Simulation in Healthcare. 2016; 11(1):1-9.
	Fritz J, Walker DM, Cohen S, Angeles G, Lamadrid-Figueroa H. Can a simulation-based training program impact the use of evidence based routine practices at birth? Results of a hospital-based cluster randomized trial in Mexico. PLoS ONE. 2017; 12(3):e0172623.

Study id	Citation
267600001	Garba MA, Mustapha GM, Rejoice C, Mustapha B, Alhaji MA, Bello IA, Hassan AE. Competence-based learning: The effectiveness of targeted resident education and clinical auditing feedback on completed death certificate accuracy rates. Nigerian Journal of Paediatrics. 2015; 42(2):132-6.
267900001	Garjani A, Salimnejad M, Shamsmohamadi M, Baghchevan V, Vahidi RG, Maleki-Dijazi N, Rezazadeh H. Effect of interactive group discussion among physicians to promote rational prescribing. Eastern Mediterranean Health Journal. 2009;15(2):408-15.
268300001	Crigler L, Boucar M, Sani K, Abdou S, Djibrina S, Saley Z. 2012. The Human Resources Collaborative: Improving Maternal and Child Care in Niger. Final Report. Bethesda (MD): University Research Co., LLC (URC); 2012. 62 p. Contract No. GHN-I-03-07-00003-00. Supported by the USAID Health Care Improvement Project.
	USAID Health Care Improvement Project. Strengthening human resources for health to improve maternal care in Niger's Tahoua region. Bethesda (MD): University Research Co., LLC (URC); 2011. 4 p. Contract No.: GHN-I-03-07-00003-00.
268400001	He P, Yuan Z, Liu Y, Li G, Lv H, Yu J, Harris MF. An evaluation of a tailored intervention on village doctors use of electronic health records. BMC Health Services Research. 2014; 14:217.
268500001	Zhang WH, Raven J, Zhang TH, Shen Y, Huang K, Long Q, Klemetti R, Temmerman M, Hemminki E and the CHIMACA study group. (2010). Implementation of interventions in the CHIMACA project. THL discussion paper 13, THL, University print, Helsinki, Finland.
	Zhang WH, Raven J, Zhang TH, Shen Y, Huang K, Long Q, Klemetti R, Temmerman M, Hemminki E, the CHIMACA study group. Implementation of interventions in the CHIMACA project. National Institute for Health and Welfare (THL), Discussion papers 13/2010. Helsinki 2010.
	Hemminki E, Long Q, Zhang WH, Wu Z, Raven J, Tao F, Yan H, Wang Y, Klemetti R, Zhang T, Regushevskaya E, Tang S. Impact of financial and educational interventions on maternity care: results of cluster randomized trials in rural China, CHIMACA. Maternal and Child Health Journal. 2013;17(2):208-21.
268500002	Zhang WH, Raven J, Zhang TH, Shen Y, Huang K, Long Q, Klemetti R, Temmerman M, Hemminki E, the CHIMACA study group. Implementation of interventions in the CHIMACA project. National Institute for Health and Welfare (THL), Discussion papers 13/2010. Helsinki 2010.
	Long Q, Huang K, Shen Y, Hemminki E. Community-based randomised financial interventions for prenatal care: lesson from implementation in two rural counties in China. https://www.thl.fi/documents/189940/1590285/Financial-intervention-30012012.pdf/33c3ac8e-df49-41cd-9545-3486c4ffa00a
	Hemminki E, Long Q, Zhang WH, Wu Z, Raven J, Tao F, Yan H, Wang Y, Klemetti R, Zhang T, Regushevskaya E, Tang S. Impact of financial and educational interventions on maternity care: results of cluster randomized trials in rural China, CHIMACA. Maternal and Child Health Journal. 2013;17(2):208-21.
268500003	Hemminki E, Long Q, Zhang WH, Wu Z, Raven J, Tao F, Yan H, Wang Y, Klemetti R, Zhang T, Regushevskaya E, Tang S. Impact of financial and educational interventions on maternity care: results of cluster randomized trials in rural China, CHIMACA. Maternal and Child Health Journal. 2013;17(2):208-21.
	Zhang WH, Raven J, Zhang TH, Shen Y, Huang K, Long Q, Klemetti R, Temmerman M, Hemminki E and the CHIMACA study group. (2010). Implementation of interventions in the CHIMACA project. THL discussion paper 13, THL, University print, Helsinki, Finland.
268500005	Zhang WH, Raven J, Zhang TH, Shen Y, Huang K, Long Q, Klemetti R, Temmerman M, Hemminki E and the CHIMACA study group. (2010). Implementation of interventions in the CHIMACA project. THL discussion paper 13, THL, University print, Helsinki, Finland.
	Hemminki E, Long Q, Zhang WH, Wu Z, Raven J, Tao F, Yan H, Wang Y, Klemetti R, Zhang T, Regushevskaya E, Tang S. Impact of financial and educational interventions on maternity care: results of cluster randomized trials in rural China, CHIMACA. Maternal and Child Health Journal. 2013;17(2):208-21.
268700001	Hengameh H, Afsaneh R, Morteza K, Hosein M, Marjan SM, Abbas E. The effect of applying direct observation of procedural skills (DOPS) on nursing students' clinical skills: A randomized clinical trial. Global Journal of Health Science. 2015; 7(7):17-21.

Study id	Citation
269400001	Huaman MA, Araujo-Castillo RV, Soto G, Neyra JM, Quispe JA, Fernandez MF, Mundaca CC, Blazes DL. Impact of two interventions on timeliness and data quality of an electronic disease surveillance system in a resource limited setting (Peru): a prospective evaluation. BMC Medical Informatics and Decision Making. 2009; 9:16
271200001	Jenkins, Rachel. Value for money of a 40 hour mental health CPD training course in Kenya for primary care health workers. 2015. 3 p.
	Jenkins R, Othieno C, Okeyo S, Kaseje D, Aruwa J, Oyug H, Bassett P, Kauye F. Short structured general mental health in service training programme in Kenya improves patient health and social outcomes but not detection of mental health problems - a pragmatic cluster randomised controlled trial. International Journal of Mental Health Systems. 2013; 7:25.
	Jenkins R, Othieno C, Okeyo S, Aruwa J, Wallcraft J, Jenkins B. Exploring the perspectives and experiences of health workers at primary health facilities in Kenya following training. International Journal of Mental Health Systems. 2013; 7:6.
	Jenkins R, Othieno C, Okeyo S, Aruwa J, Kingora J, Jenkins B. Health systems challenges to integration of mental health delivery in primary care in Kenya - perspectives of primary care health workers. BMC Health Services Research. 2013; 13:368.
	Othieno C, Jenkins R, Okeyo S, Aruwa J, Wallcraft J, Jenkins B. Perspectives and concerns of clients at primary health care facilities involved in evaluation of a national mental health training programme for primary care in Kenya. International Journal of Mental Health Systems. 2013; 7(5):1-7.
	Jenkins R, Kiima D, Njenga F, Okonji M, Kingora J, Kathuku D, Lock S. Integration of mental health into primary care in Kenya. World Psychiatry. 2010; 9(2): 118-20.
	Jenkins R, Kiima D, Okonji M, Njenga F, Kingora J, Lock S. Integration of mental health into primary care and community health working in Kenya: context, rationale, coverage and sustainability. Mental Health in Family Medicine. 2010; 7(1): 37-47.
271800001	Jere DL, Kaponda CPN, Chimwaza A, Crittenden KS, Kachingwe ST, McCreary L, Norr JL, Norr K. Improving universal precautions and client teaching for rural health workers: a peer-group intervention. AIDS Care. 2010; 22(5):649-57.
	Norr K, Kaponda CPN, Dancy BL, Jere DL, Kachingwe SI, Kafulafula U, McCreary LL, Mbeba MM, Norr JL, Talashek ML. A primary health care intervention to mobilize health workers for HIV prevention in Malawi. Primary Health Care Research and Development. 2006; 7:318-30.
	Talashek ML, Kaponda CPN, Jere DL, Kafulafula U, Mbeba MM, McCreary LL, Norr K. Identifying what rural health workers in Malawi need to become HIV prevention leaders. Journal of the Association of Nurses in AIDS Care. 2007; 18(4):41-50.
	Mbeba M, Kaponda CPN, Jere DL, Kachingwe SI, Crittenden KS, McCreary LL, Norr JL, Norr KF. Peer group intervention reduces personal HIV risk for Malawian health workers. Journal of Nursing Scholarship. 2011; 43(1):72-81.
	Kaponda CPN, Jere DL, Chimango JL, Chimwaza AF, Crittenden KS, Kachingwe SI, McCreary LL, Norr JL, Norr KF. Impacts of a peer-group intervention on HIV-related knowledge, attitudes, and personal behaviors for urban hospital workers in Malawi. Journal of the Association of Nurses in AIDS Care. 2009; 20(3):230-42.
274800001	Teng CL, Achike FI, Phua KL, Nurjahan MI, Mastura I, Asiah HN, Mariam AM, Narayanan S, Norsiah A, Sabariah I, Shariah I, Rokiah KS, Zubaidah MA, Koh CN, Rosnah I. Modifying antibiotic prescribing: the effectiveness of academic detailing plus information leaflet in a Malaysian primary care setting. The Medical Journal of Malaysia. 2006; 61(3):323-31.
	Mastura I, Teng CL. The effect of 'group detailing' on drug prescribing in primary care. The Medical Journal of Malaysia. 2008; 63(4): 315-8.
275900001	Nanda P, Mishra A, Mukherjee S, Barua A, Mehl GL, Venkatraman CM. A study to evaluate the effectiveness of WHO tools--Orientation Programme on Adolescent Health for Health Care Providers and Adolescent Job Aid--in improving the quality of health services provided by health workers to their female adolescent clients in India. Geneva: WHO Press; 2012. 74 p. Report of a WHO, UNFPA, UNICEF Study Group

Study id	Citation
276000001	Nguyen HT, Pham HT, Vo DK, Nguyen TD, van den Heuvel ER, Haaijer-Ruskamp FM, Taxis K. The effect of a clinical pharmacist-led training programme on intravenous medication errors: a controlled before and after study. BMJ Quality and Safety. 2014; 23(4): 319-24.
	Nguyen HT, Nguyen TD, van den Heuvel ER, Haaijer-Ruskamp FM, Taxis K. Errors in medicines preparation and administration in Vietnamese hospitals. Poster session presented at: 18th Congress of the European Association of Hospital Pharmacists; 2013 Mar 13-15; Paris, France.
	Nguyen HT, Nguyen TD, van den Heuvel ER, Haaijer-Ruskamp FM, Taxis K. Medication errors in Vietnamese hospitals: prevalence, potential outcome and associated factors. PLoS One. 2015 Sep 18;10(9).
276100001	Bin Nisar Y, Hafeez A, Zafar S, Southall DP. Impact of essential surgical skills with an emphasis on emergency maternal, neonatal and child health training on the practice of doctors: a cluster randomised controlled trial in Pakistan. Resuscitation. 2011; 82(8):1047-52.
278600001	Rahnavard Z, Nodeh ZH, Hossein L. Effectiveness of clinical teaching associate model in nursing education: results from a developing county. Contemporary Nurse. 2013; 45 (2): 174-81.
278800001	Reynolds HW, Toroitich-Ruto C, Nasution M, Beaston-Blaakman A, Janowitz B. Effectiveness of training supervisors to improve reproductive health quality of care: a cluster-randomized trial in Kenya. Health Policy Planning. 2008; 23(1): 56-66.
279300001	Silva JM, Stein AT, Schunemann HJ, Bordin R, Kuchenbecker R, de Lourdes Drachler M. Academic detailing and adherence to guidelines for Group B streptococci prenatal screening: a randomized controlled trial. BMC Pregnancy and Childbirth. 2013; 13:68.
281600001	Sánchez-Mendiola M, Kieffer-Escobar LF, Marín-Beltrán S, Downing SM, Schwartz A. Teaching of evidence-based medicine to medical students in Mexico: a randomized controlled trial. BMC Medical Education. 2012; 12:107.
281700001	Varghese NC, Macaden L, Premkumar B, Mathews P, Kumar S. Delirium in older people in hospital: an education programme. British Journal of Nursing. 2014; 23(13):704-9.
283700001	Weaver MR, Pillay E, Jed SL, de Kadt J, Galagan S, Gilvydis J, Marumo E, Mawandia S, Naidoo E, Owens T, Prongay V, O'Malley G. Three methods of delivering clinic-based training on syndromic management of sexually transmitted diseases in South Africa: a pilot study. Sexually Transmitted Infections. 2016; 92(2):135-41.
284200001	Zhang CH, Hsu L, Zou BR, Li JF, Wang HY, Huang J. Effects of a pain education program on nurses' pain knowledge, attitudes and pain assessment practices in China. Journal of Pain and Symptom Management. 2008; 36(6): 616-27.
	Zhang CH, Xu LH, Zhu XP, Wang HY, Huang Y, Li JF, Zheng L. Research about the effect of pain-related education program on nurses' attitude, knowledge and assessment practices of pains. Chinese Journal of Practical Nursing. 2010; 26 (2A):30-33. [Chinese]
287700001	Kyriacos U, Jelsma J, James M, Jordan S. Early warning scoring systems versus standard observations charts for wards in South Africa: a cluster randomized controlled trial. Trials. 2015; 20;16:103.
	Kyriacos U, Jelsma J, James M, Jordan S. Monitoring vital signs: development of a modified early warning scoring (MEWS) system for general wards in a developing country. PLoS One. 2014, 24;9(1):e87073.
287800001	Lourenço C, Kandula D, Haidula L, Ward A, Cohen JM. Strengthening malaria diagnosis and appropriate treatment in Namibia: a test of case management training interventions in Kavango Region. Malaria Journal. 2014; 13:508.
287900001	Mahomed OH, Naidoo S, Asmall S, Taylor M. Improving the quality of nurse clinical documentation for chronic patients at primary care clinics: a multifaceted intervention. Curationis. 2015; 38 (1).
289400001	Yousefi H, Nahidian M, Sabouhi F. Reviewing the effects of an educational program about sepsis care on knowledge, attitude, and practice of nurses in intensive care units. Iranian Journal of Nursing and Midwifery Research. 2012; 17 (2 Suppl 1):S91-5.
289600001	Zwarenstein M, Fairall LR, Lombard C, Mayers P, Bheekie A, English RG, Lewin S, Bachmann MO, Bateman E. Outreach education for integration of HIV/AIDS care, antiretroviral treatment, and tuberculosis care in primary care clinics in South Africa: PALSA PLUS pragmatic cluster randomised trial. British Medical Journal. 2011; 342:d2022.

Study id	Citation
292600001	Mengistu B, Karim AM, Eniyew A, Yitabrek A, Eniyew A, Tsegaye S, Muluye F, Tesfaye H, Demeke B, Marsh DR. Effect of performance review and clinical mentoring meetings (PRCMM) on recording of community case management by health extension workers in Ethiopia. Ethiopian Medical Journal. 2014; 52 Suppl 3:73-81.
292700001	N'Guessan J, Traore V, Boucar M, Ackah A, Dosso Y, Kouassi V, Kablan F. Results from the pilot phase of an ART/PMTCT improvement collaborative in Cote d'Ivoire. Technical Report. Bethesda, (MD): University Research Co., LLC (URC); 2011 13p. Contract Nos.: GHN-I-01-07-00003-00 and GHN-I-03-07-00003-00. Supported by the USAID Health Care Improvement Project.
	N'Guessan J, Franco L, Ackah A, Kouassi VK, Gondwe T. Effects of collaborative improvement on PMTCT and ART indicators in Cote d'Ivoire: a comparative study. Bethesda, (MD): University Research Co., LLC (URC); 2011 1p. Contract Nos.: GHN-I-01-07-00003-00 and GHN-I-03-07-00003-00. Supported by the USAID Health Care Improvement Project.
293500001	Vukovic M, Gvozdenovic BS, Rankovic M, McCormick BP, Vukovic DD, Gvozdenovic BD, Kastratovic DA, Markovic SZ, Ilic M, Jakovljevic MB. Can didactic continuing education improve clinical decision making and reduce cost of quality? Evidence from a case study. Journal of Continuing Education in the Health Professions. 2015; 35(2):109-18.
296600001	Rambaud-Althaus C, Shao A, Samaka J, Swai N, Perri S, Kahama-Maro J, Mitchell M, D'Acremont V, Genton B. Performance of health workers using an electronic algorithm for the management of childhood illness in Tanzania: a pilot implementation study. The American Journal of Tropical Medicine and Hygiene. 2017; 96(1):249-257.
	Shao AF, Rambaud-Althaus C, Swai N, Kahama-Maro J, Genton B, D'Acremont V, Pfeiffer C. Can smartphones and tablets improve the management of childhood illness in Tanzania? A qualitative study from a primary health care worker's perspective. BMC Health Services Research. 2015; 15:135.
299490102	Westercamp N, Staedke S, Hutchinson E, Naiga S, Nabirye C, Taaka L, Maiteki C, Kigozi S, Okiring JM, Dorsey G, Rowe AK. Effectiveness and sustainability of a collaborative improvement method to increase the quality of routine malaria surveillance data in Kayunga District, Uganda. Proceedings of the 66th Annual Meeting of the American Society of Tropical Medicine and Hygiene; 2017 Nov 5-9; Baltimore, MD.
	Fatuma A. The Republic of Uganda, Kayunga District Local Government: 3-year district development plan, 2010/2011 - 2012/2013. 2010 Apr 28. 233 p.
	Hutchinson E, Nayiga S, Nabirye C, Taaka L, Westercamp N, Rowe AK, Staedke SG. Opening the 'Black Box' of collaborative improvement: a qualitative evaluation of a pilot intervention to improve quality of surveillance data in public health centres in Uganda. 2017. 35 p.
300800001	Chitashvili T, Cherkezishvili E, Broughton E, Chkhaidze I, Shengelia N, Hill K, Massoud MR, Ruadze E. Improving antibiotic prescription practices for pediatric respiratory tract infections in Georgia. Forthcoming (submitted for publication to Lancet Infectious Diseases); 2017. 22p.
	Chitashvili T. Rationale for improving integrated service delivery: reduced cost and improved care in Georgia. International Journal of Integrated Care. 2015; 15(8). A paper presented at the 3rd World Congress on Integrated Care; 2015; Nov 20-21, Mexico City. 25 slides.
	Chitashvili T. Scaling Up, sustaining and institutionalizing better health care in Georgia: results and strategic recommendations from USAID support for improving quality of priority clinical conditions during 2012-2015. Technical Report. Bethesda (MD): USAID ASSIST Project: University Research Co., LLC (URC). 2015; 17 p. Contract No.: GHN-I-03-07-00003-00 and Cooperative Agreement No.: AID-OAA-A-12-00101.
	Chitashvili T. Addressing rational use of medication in pediatric patients with respiratory tract infections (RTI) through improvement collaborative in Georgia. A poster presented at the third Global Symposium on Health Systems Research; 2014; Sep 30-Oct 3, Cape Town, South Africa.

Study id	Citation
	USAID ASSIST Project: University Research Co., LLC (URC) (Bethesda, MD). USAID Applying Science to Strengthen and Improve Systems (ASSIST) Project. Georgia Country Report FY14; 2014. 17 p. Cooperative Agreement No.: AID-OAA-A-12-00101.
	Chitashvili T, Cherkezishvili E. Improving quality of care for respiratory tract infections in children: the role of capacity building and coaching in supporting one multi-facility improvement team in Samtredia district, Georgia. Submitted to a journal for publication. 2017, 16p.
302900001	Barceló A, Cafiero E, de Boer M, Mesa AE, Lopez MG, Jiménez RA, Esqueda AL,Martinez JA, Holguin EM, Meiners M, Bonfil GM, Ramirez SN, Flores EP, Robles S.Using collaborative learning to improve diabetes care and outcomes: the VIDA project. Primary Care Diabetes. 2010; 4(3):145-53.

Table B. Sample size information for included studies

**Study level**				
	**Study design**			
**Strategy**	**ITS studies only**	**Non-ITS studies with at least 2 follow-up points**	**Non-ITS studies with only 1 follow-up point**	**Total no. of studies**
Training alone	2	15	48	65
Supervision alone	3	2	11	16
Training plus supervision	0	5	17	22
Group problem solving	8	0	4	12
Group problem solving plus training	4	0	0	4
Total number of studies	17	20*	77*	114*

**Outcome level†**				
	**Study design**			
**Strategy**	**ITS studies only**	**Non-ITS studies with at least 2 follow-up points**	**Non-ITS studies with only 1 follow-up point**	**Total no. of outcomes**
Training alone	4	75	357	436
Supervision alone	5	3	43	51
Training plus supervision	0	43	66	109
Group problem solving	15	0	16	31
Group problem solving plus training	10	0	0	10
Total number of outcomes	34	121	482	637

**Effect size level**				
	**Study design**			
**Strategy**	**ITS studies only**	**Non-ITS studies with at least 2 follow-up points**	**Non-ITS studies with only 1 follow-up point**	**Total no. of effect sizes**
Training alone	29	163	357	549
Supervision alone	39	8	43	90
Training plus supervision	0	90	66	156
Group problem solving	230	0	16	246
Group problem solving plus training	199	0	0	199
Total number of effect sizes	497	261	482	1240

* The sum of the individual rows is larger than the overall total because some studies had multiple study arms and could appear more than once in a column. One non-ITS study with at least 2 follow-up points had a “training alone” arm and a “training plus supervision” arm, and one non-ITS study with at least 2 follow-up points had a “supervision alone” arm and two “training plus supervision” arms. Two non-ITS

studies with only 1 follow-up point had a “training alone” arm and a “training plus supervision” arm, and one non-ITS study with only 1 follow-up point had a “supervision alone” arm and two “training plus supervision” arms.

† In this table, outcomes are counted separately each time they are used by a unique study comparison. For example, if the outcome “percentage of patients correctly treated” is used in one study with one study comparison (i.e., an intervention arm versus a control arm), then the study would be counted as having one outcome. If the same outcome is used in a different study that had two study comparisons (i.e., two intervention arms, and a control arm), then the study would be counted as having two outcomes. This approach was used because the outcome counts reflect the number of lines in the results graphs. Each line corresponds to a series of effect sizes for a given outcome from a given study comparison.

Arsenault C, *et al. BMJ Qual Saf* 2022; 31:123–133. doi: 10.1136/bmjqs-2020-011717

Table C. Attributes of included studies

Study attribute	ITS + non-ITS studies with >2 follow-up measurements (primary analyses) (N = 37)	ITS + all non-ITS studies (sensitivity analyses) (N = 114)
Year of publication		
2010 or later	17 (46.0)	43 (37.7)
2000–2009	16 (43.2)	50 (43.9)
1990–1999	4 (10.8)	21 (18.4)
Study design		
Pre-post study with randomized controls	16 (43.2)	45 (39.5)
Interrupted time series with no controls	16 (43.2)	16 (14.0)
Pre-post study with non-randomized controls	4 (10.8)	32 (28.1)
Interrupted time series with randomized controls	1 (2.8)	1 (0.9)
Post-only study with randomized controls	0 (0)	20 (17.5)
Places where services were delivered (multiple responses allowed)		
Outpatient health facility	22 (59.5)	73 (64.4)
Hospital outpatient department	13 (35.1)	31 (27.2)
Hospital inpatient wards	11 (29.7)	31 (27.2)
Non-hospital health facility inpatient ward	3 (8.1)	3 (2.6)
Household or community setting	2 (5.4)	5 (4.4)
Pharmacy	2 (5.4)	5 (4.4)
Drug shop	1 (2.7)	2 (1.8)
School	0	2 (1.8)
Site in transit to hospital or health facility	0	1 (0.9)
Other outpatient setting	1 (2.7)	2 (1.8)
Who owns or operates the place where services were delivered (multiple responses allowed per study)		
Public or government	32 (86.5)	97 (85.1)
Private, for profit	4 (10.8)	12 (10.5)
Private, not for profit	4 (10.8)	7 (6.1)
Community	2 (5.4)	7 (6.1)
Private, profit status unknown or not reported	0	4 (3.5)
Other	1 (2.7)	1 (0.9)
Unclear or not reported	3 (8.1)	7 (6.1)
Economy of country where study was done		
Low income	22 (59.5)	45 (39.5)
Lower-middle income	7 (18.9)	35 (30.7)
Upper-middle income	8 (21.6)	33 (29.0)

**Study attribute**	**ITS + non-ITS studies with >2 follow-up measurements (primary analyses) (N = 37)**	**ITS + all non-ITS studies (sensitivity analyses) (N = 114)**
Combination of lower-middle and upper-middle income	0	1 (0.9)
WHO region where study was conducted		
Africa	17 (46.0)	40 (35.1)
Southeast Asia	7 (18.9)	26 (22.8)
Americas	4 (10.8)	16 (14.0)
Western Pacific	2 (5.4)	15 (13.2)
Eastern Mediterranean	3 (8.1)	12 (10.5)
Europe	4 (10.8)	5 (4.4)
Risk of bias		
Low	6 (16.2)	16 (14.0)
Moderate	7 (18.9)	29 (25.4)
High	11 (29.7)	39 (34.2)
Very high	13 (35.1)	30 (26.3)
Total number of outcomes*	155	637
Treatment	57 (36.8)	232 (36.4)
Counseling	37 (23.9)	153 (24.0)
Patient assessment	26 (16.8)	99 (15.5)
Case management	15 (9.7)	60 (9.4)
Documentation	11 (7.1)	38 (6.0)
Referral	4 (2.6)	13 (2.0)
Universal precautions	2 (1.3)	13 (2.0)
Diagnosis	1 (0.6)	11 (1.7)
Vaccination	0 (0)	1 (0.2)
Other practice	2 (1.3)	17 (2.7)
Total number of comparisons		
Training alone	17	70
Group in-service training alone	15 (88.2)	56 (80.0)
Academic detailing alone	1 (5.9)	7 (10.0)
Group in-service training + peer-to-peer education	1 (5.9)	3 (4.3)
Group pre-service training alone	0	2 (2.9)
Peer-to-peer education alone	0	1 (1.4)
Group in-service training + self-study	0	1 (1.4)
Supervision alone	5	16
Audit with in-person feedback alone	4 (80.0)	6 (37.5)
Audit with in-person feedback + peer review	1 (20.0)	1 (6.2)
Routine supervision alone	0	9 (56.3)

Study attribute	ITS + non-ITS studies with >2 follow-up measurements (primary analyses) (N = 37)	ITS + all non-ITS studies (sensitivity analyses) (N = 114)
Training plus supervision	6	25
Group in-service training + routine supervision	3 (50.0)	11 (44.0)
Group in-service training + benchmarking + audit with written feedback	1 (16.7)	1 (4.0)
Group in-service training + audit with in-person and written feedback	1 (16.7)	3 (12.0)
Academic detailing + audit with in-person and written feedback	1 (16.7)	2 (8.0)
Group in-service training + audit with in-person feedback	0	2 (8.0)
Pre-service training + routine supervision	0	1 (4.0)
Academic detailing + routine supervision + non-supervisor support	0	1 (4.0)
Group in-service training + audit with in-person feedback + peer review	0	1 (4.0)
Group in-service training + non-supervisor support	0	1 (4.0)
Group in-service training + benchmarking + routine supervision + audit with in-person and written feedback	0	1 (4.0)
Group in-service training + peer-to-peer education + routine supervision	0	1 (4.0)
Group problem solving alone	8	12
Improvement collaborative	7 (87.5)	9 (75.0)
Continuous quality improvement	1 (12.5)	2 (16.7)
Team-based problem solving	0	1 (8.3)
Group problem solving plus training	4	4
Improvement collaborative + group in-service training	4 (100)	4 (100)

* In this table, outcomes are counted separately each time they are used by a unique study comparison. For example, if the outcome “percentage of patients correctly treated” is used in one study with one study comparison (i.e., an intervention arm versus a control arm), then the study would be counted as having one outcome. If the same outcome is used in a different study that had two study comparisons (i.e., two intervention arms, and a control arm), then the study would be counted as having two outcomes. This approach was used because the outcome counts reflect the number of lines in the results graphs. Each line corresponds to a series of effect sizes for a given outcome from a given study comparison.

Table D. Sample size information for included studies, stratified by training and supervision categories

**Study level**				
	**Study design**			
**Strategy**	**ITS studies only**	**Non-ITS studies with at least 2 follow-up points**	**Non-ITS studies with only 1 follow-up point**	**Total no. of studies**
Category of "training alone" strategy				
One-time training	2	9	42	53
Interrupted training	0	6	6	12
Category of "supervision alone" strategy				
One-time supervision	1	0	2	3
Ongoing supervision	2	2	9	13
Category of "training and supervision" strategy				
One-time training and one-time supervision	0	1	5	6
One-time training and ongoing supervision	0	4	12	16

**Outcome level***				
	**Study design**			
**Strategy**	**ITS studies only**	**Non-ITS studies with at least 2 follow-up points**	**Non-ITS studies with only 1 follow-up point**	**Total no. of outcomes**
Category of "training alone" strategy				
One-time training	4	55	303	362
Interrupted training	0	20	54	74
Category of "supervision alone" strategy				
One-time supervision	1	0	18	19
Ongoing supervision	4	3	25	32
Category of "training and supervision" strategy				
One-time training and one-time supervision	0	35	41	76
One-time training and ongoing supervision	0	8	25	33

Arsenault C, *et al. BMJ Qual Saf* 2022; 31:123–133. doi: 10.1136/bmjqs-2020-011717

**Effect size level**				
	**Study design**			
**Strategy**	**ITS studies only**	**Non-ITS studies with at least 2 follow-up points**	**Non-ITS studies with only 1 follow-up point**	**Total no. of effect sizes**
Category of "training alone" strategy				
One-time training	29	116	303	448
Interrupted training	0	47	54	101
Category of "supervision alone" strategy				
One-time supervision	6	0	18	24
Ongoing supervision	33	8	25	66
Category of "training and supervision" strategy				
One-time training and one-time supervision	0	70	41	111
One-time training and ongoing supervision	0	20	25	45

* In this table, outcomes are counted separately each time they are used by a unique study comparison. For example, if the outcome "percentage of patients correctly treated" is used in one study with one study comparison (i.e., an intervention arm versus a control arm), then the study would be counted as having one outcome. If the same outcome is used in a different study that had two study comparisons (i.e., two intervention arms, and a control arm), then the study would be counted as having two outcomes. This approach was used because the outcome counts reflect the number of lines in the results graphs. Each line corresponds to a series of effect sizes for a given outcome from a given study comparison.

Arsenault C, *et al. BMJ Qual Saf* 2022; 31:123–133. doi: 10.1136/bmjqs-2020-011717

Table E. Association between follow-up time and strategy effectiveness, stratified by training and supervision categories: estimates from random intercept linear regression models*

	ITS + non-ITS studies with at least 2 follow-up points					
	**N effect sizes**	**N studies**	**Coeff**	**p-value**	**95% CI**	
Category of "training alone" strategy						
One-time training	145	11	-0.18	0.099	-0.39	0.03
Interrupted training	47	6	-0.66	0.094	-1.43	0.11
Category of "supervision alone" strategy						
One-time supervision	6	1	NA	NA	NA	NA
Ongoing supervision	41	4	0.85	<0.0001	0.64	1.05
Category of "training and supervision" strategy						
One-time training and one-time supervision	70	1	NA	NA	NA	NA
One-time training and ongoing supervision	20	3	-0.77	<0.0001	-0.86	-0.69
	**All studies**					
	**N effect sizes**	**N studies**	**Coeff**	**p-value**	**95% CI**	
Category of "training alone" strategy						
One-time training	448	53	-0.34	0.007	-0.58	-0.09
Interrupted training	101	12	-0.59	0.020	-1.08	-0.09
Category of "supervision alone" strategy						
One-time supervision	24	3	2.97	<0.0001	1.70	4.24
Ongoing supervision	66	13	0.81	<0.0001	0.55	1.07
Category of "training and supervision" strategy						
One-time training and one-time supervision	111	6	-0.44	0.387	-1.44	0.56
One-time training and ongoing supervision	45	16	-0.59	0.068	-1.23	0.04

95% CI = 95 % confidence interval.
Coeff = The model coefficient, which is the mean percentage-point change in health care provider practice outcomes per month.
NA = Not applicable. Modeling was done only for strategy categories with $\geq$3 studies.

*All models are adjusted for health care provider's baseline performance.

Figure A. Effectiveness of “training alone” over time by study design

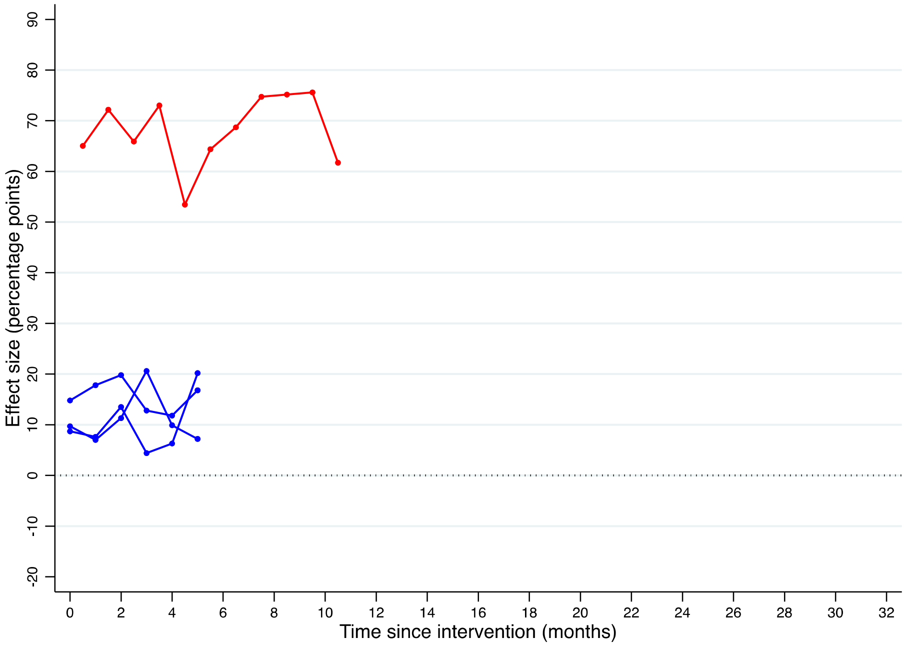

ITS studies only

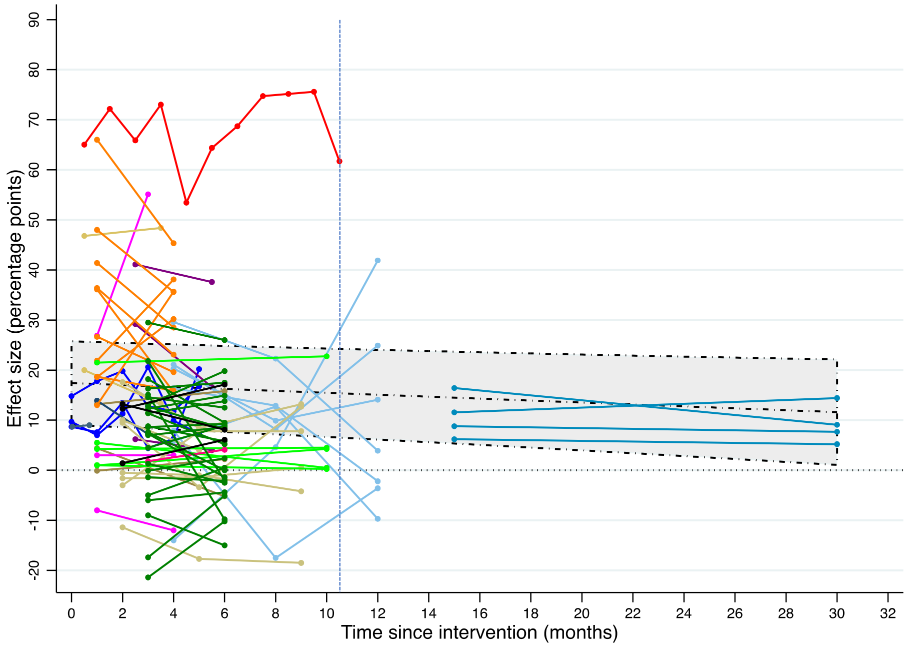

ITS and non-ITS studies with at least 2 follow-up measurements

Arsenault C, *et al. BMJ Qual Saf* 2022; 31:123–133. doi: 10.1136/bmjqs-2020-011717

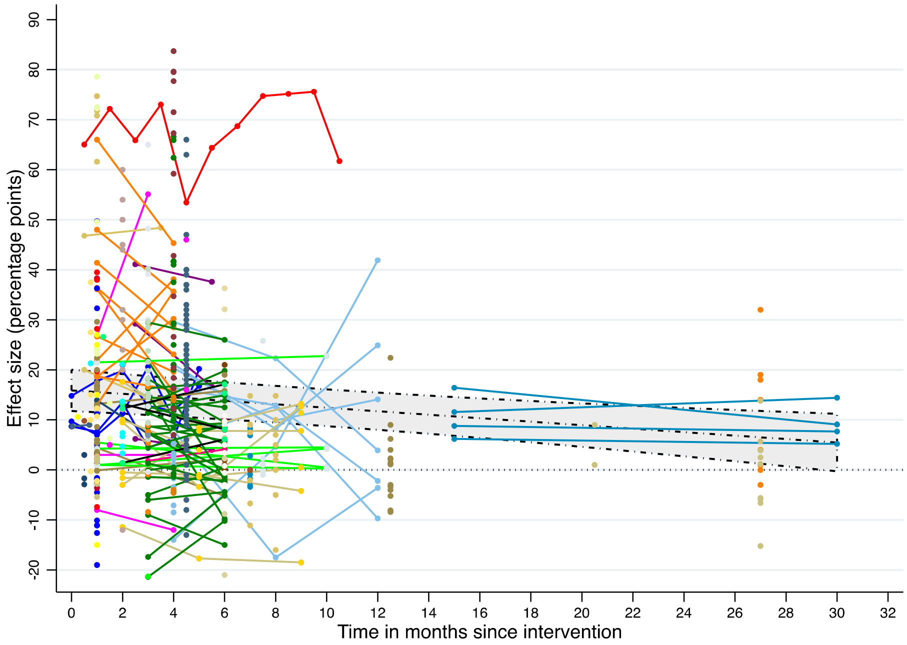

All studies

Notes:
Each study's data are represented by one color, with time-specific effect sizes from the same outcome connected with a line.
The middle dotted line in the shaded area indicates the predicted effect size at each time point based on the linear regression model.
The upper and lower dotted lines in the shaded area indicate the 95% confidence band around the predicted effect sizes.
The blue vertical line indicates the latest follow-up time point involving at least 3 studies (only applies to the dataset with ITS and non-ITS studies with at least 2 follow-up measurements).

Figure B. Effectiveness of "supervision alone" over time by study design

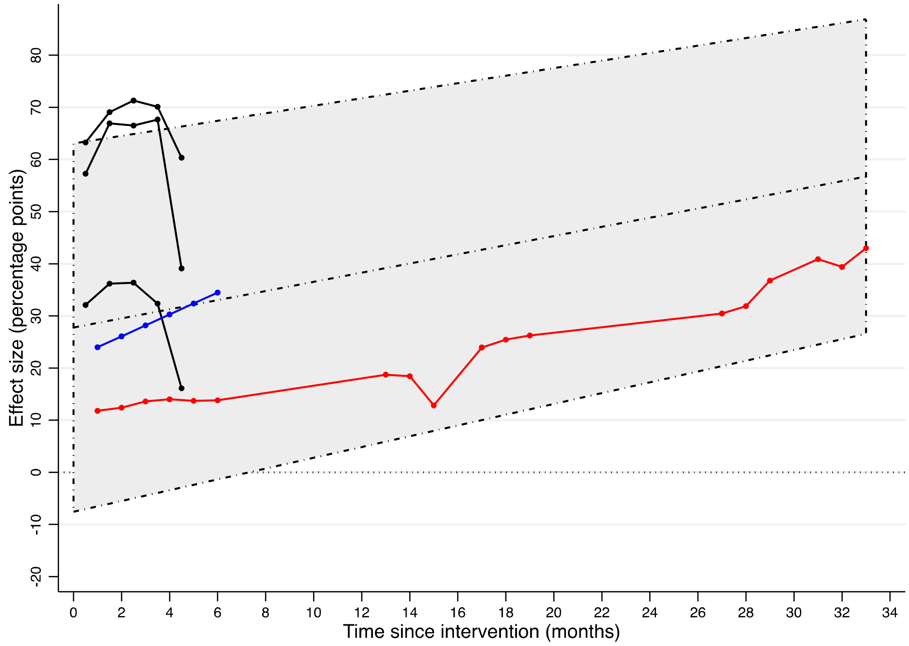

ITS studies only

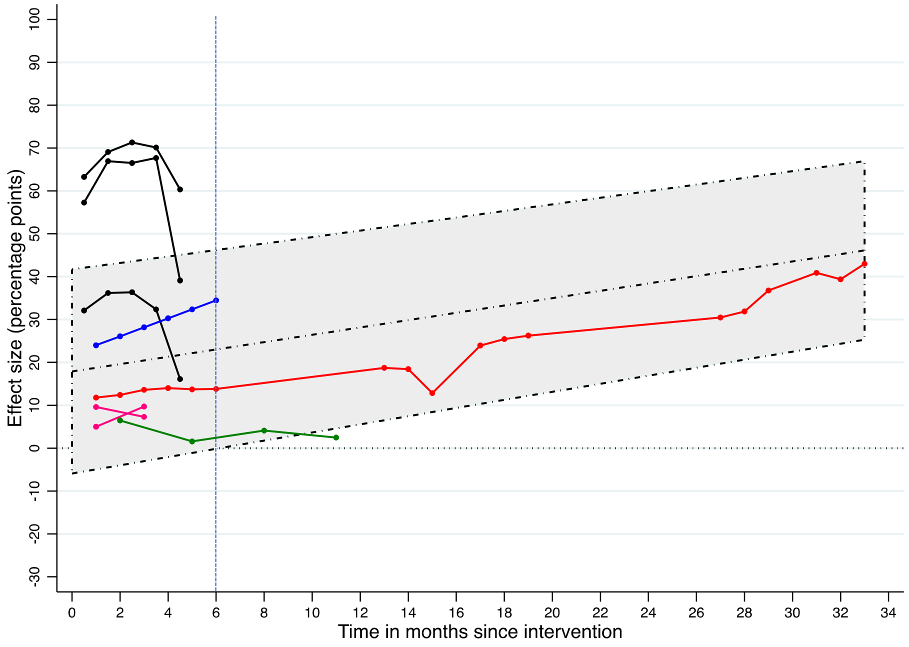

ITS and non-ITS studies with at least 2 follow-up measurements

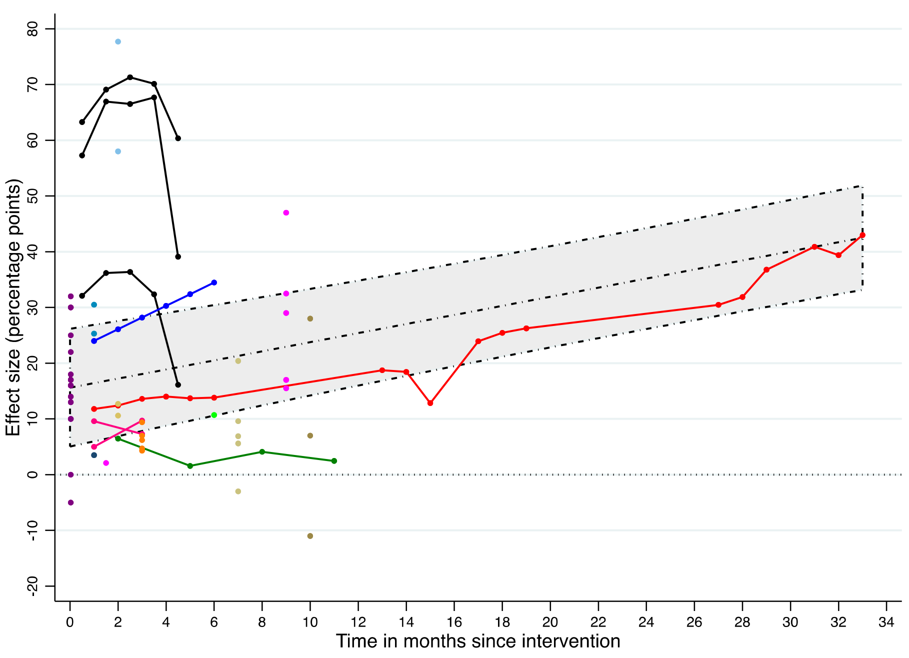

All studies

Notes:
Each study’s data are represented by one color, with time-specific effect sizes from the same outcome connected with a line.
The middle dotted line in the shaded area indicates the predicted effect size at each time point based on the linear regression model.
The upper and lower dotted lines in the shaded area indicate the 95% confidence band around the predicted effect sizes.
The blue vertical line indicates the latest follow-up time point involving at least 3 studies (only applies to the dataset with ITS and non-ITS studies with at least 2 follow-up measurements).

Figure C. Effectiveness of "training plus supervision" over time by study design

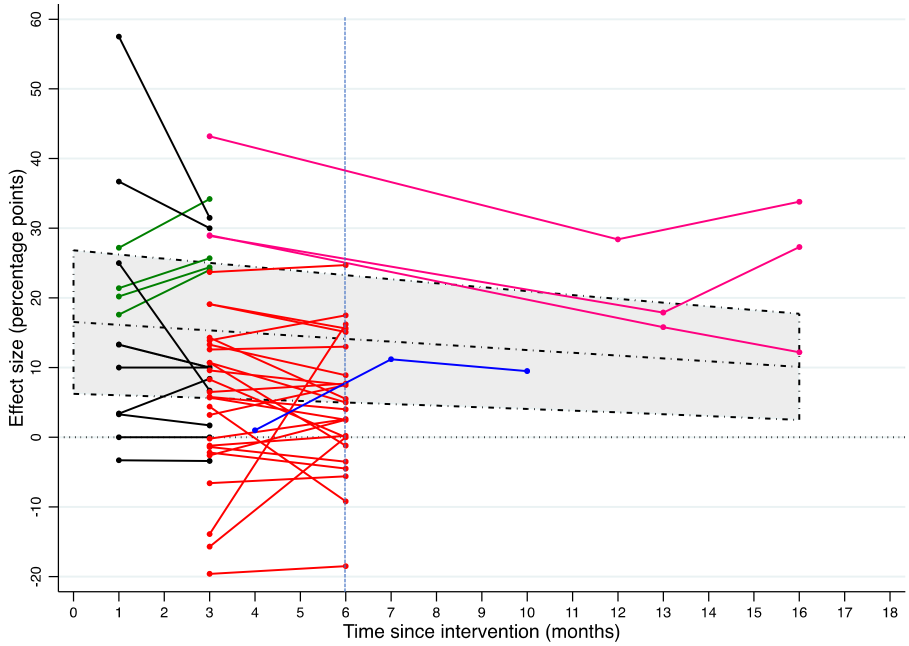

Non-ITS studies with at least 2 follow-up measurements

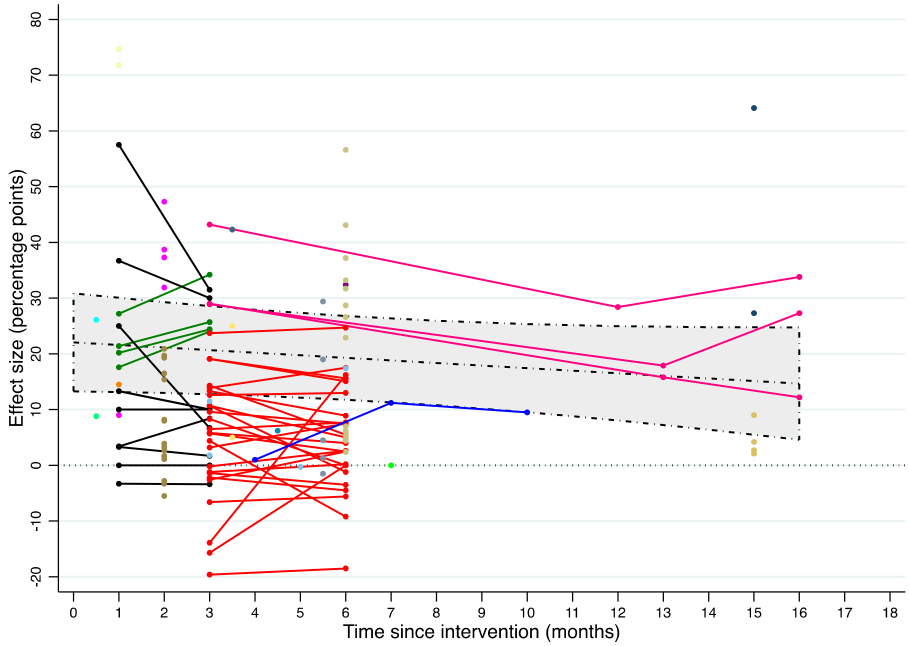

All studies

Notes:
Each study's data are represented by one color, with time-specific effect sizes from the same outcome connected with a line.

Arsenault C, *et al. BMJ Qual Saf* 2022; 31:123–133. doi: 10.1136/bmjqs-2020-011717

The middle dotted line in the shaded area indicates the predicted effect size at each time point based on the linear regression model.
The upper and lower dotted lines in the shaded area indicate the 95% confidence band around the predicted effect sizes.
The blue vertical line indicates the latest follow-up time point involving at least 3 studies (only applies to the dataset with non-ITS studies with at least 2 follow-up measurements).

Figure D. Effectiveness of "group problem solving alone" over time by study design

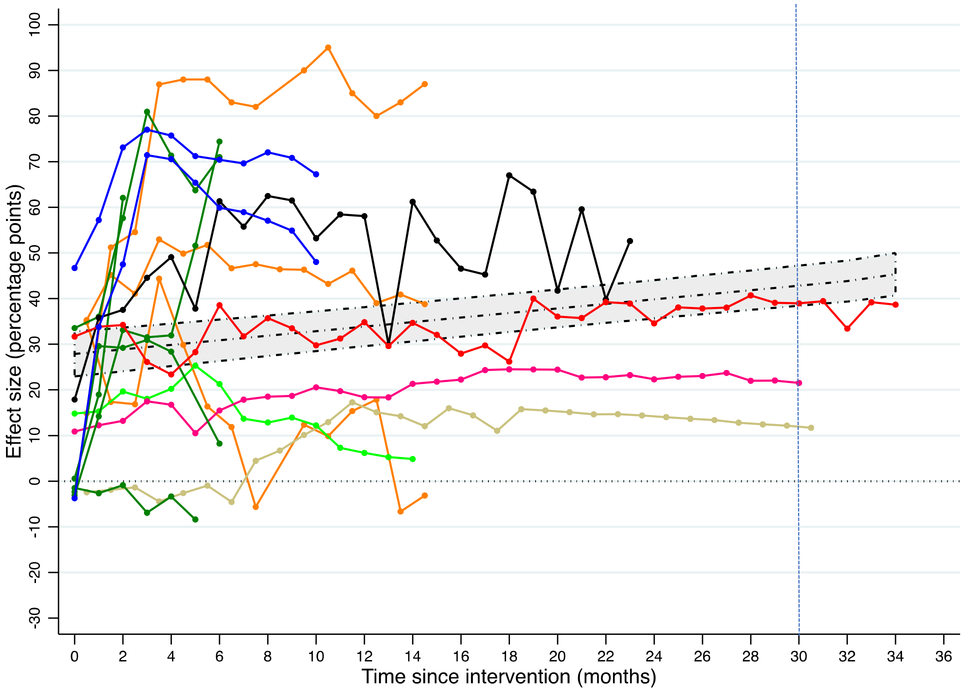

ITS studies only

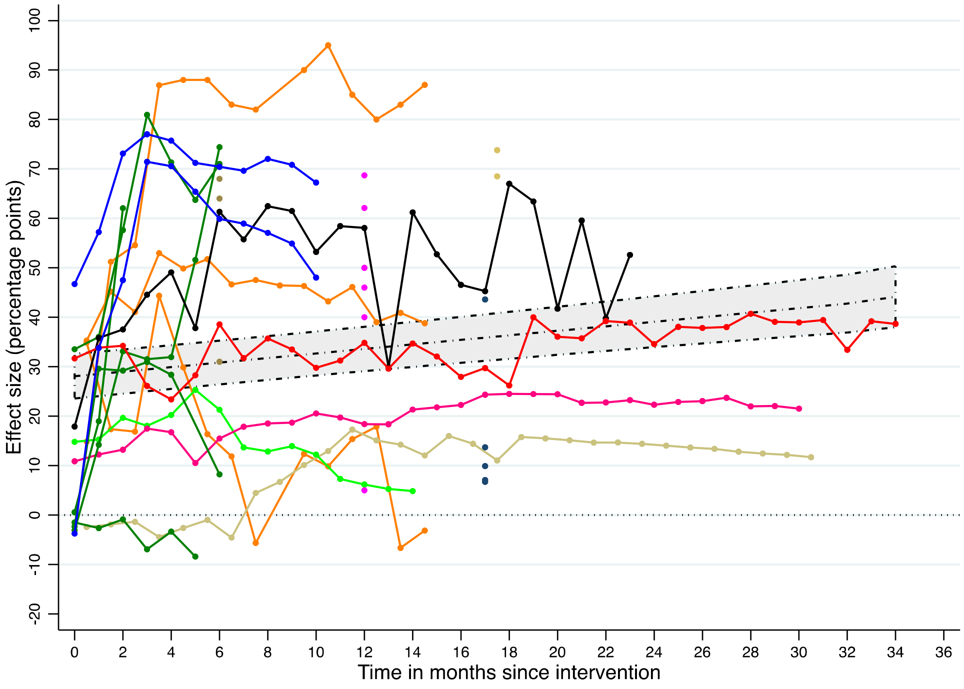

All studies

Notes:

Arsenault C, *et al. BMJ Qual Saf* 2022; 31:123–133. doi: 10.1136/bmjqs-2020-011717

Each study's data are represented by one color, with time-specific effect sizes from the same outcome connected with a line.
The middle dotted line in the shaded area indicates the predicted effect size at each time point based on the linear regression model.
The upper and lower dotted lines in the shaded area indicate the 95% confidence band around the predicted effect sizes.
The blue vertical line indicates the latest follow-up time point involving at least 3 studies (only applies to the dataset with ITS studies).

Arsenault C, *et al. BMJ Qual Saf* 2022; 31:123–133. doi: 10.1136/bmjqs-2020-011717

Figure E. Effectiveness of “group problem solving plus training” over time by study design

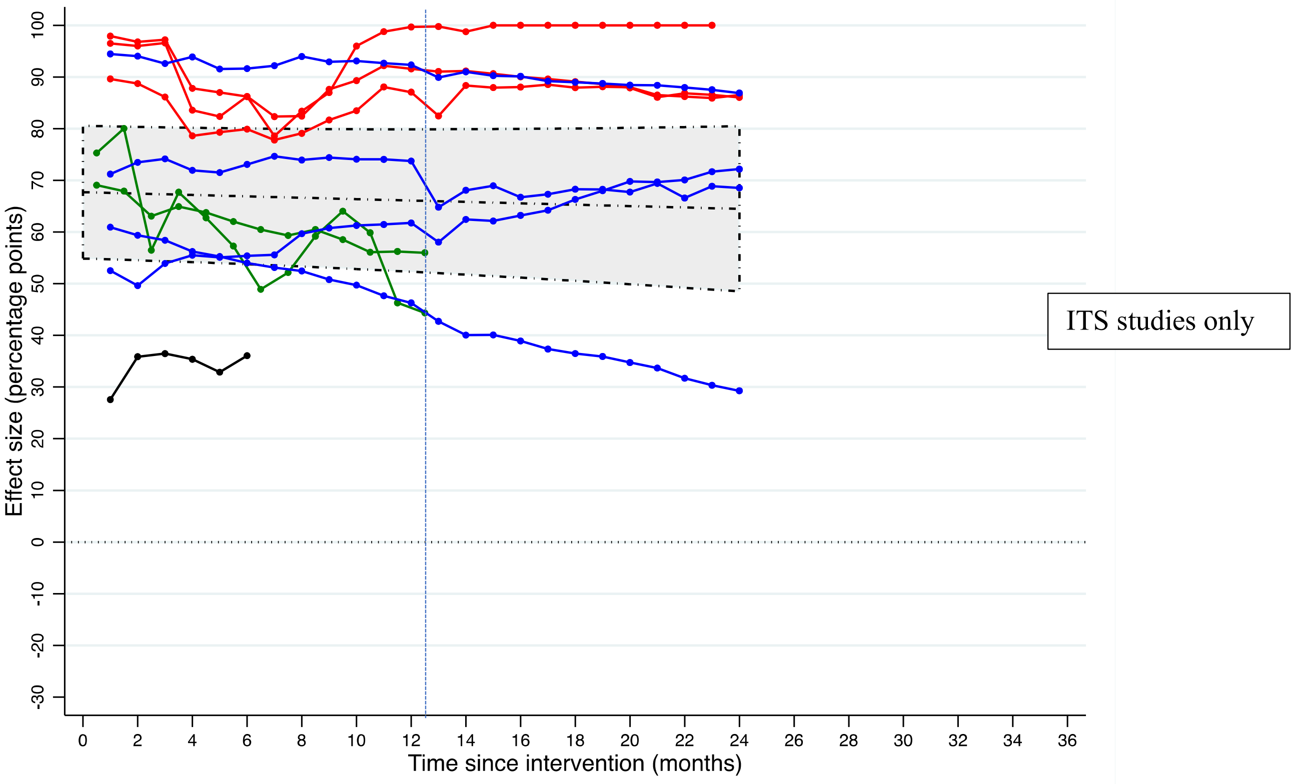

Notes:
Each study’s data are represented by one color, with time-specific effect sizes from the same outcome connected with a line.
The middle dotted line in the shaded area indicates the predicted effect size at each time point based on the linear regression model.
The upper and lower dotted lines in the shaded area indicate the 95% confidence band around the predicted effect sizes.
The blue vertical line indicates the latest follow-up time point involving at least 3 studies.

Figure F. Distribution of effect size follow-up times, in months, for each strategy: results from interrupted time series studies plus non-interrupted time series studies with at least two follow-up measurements

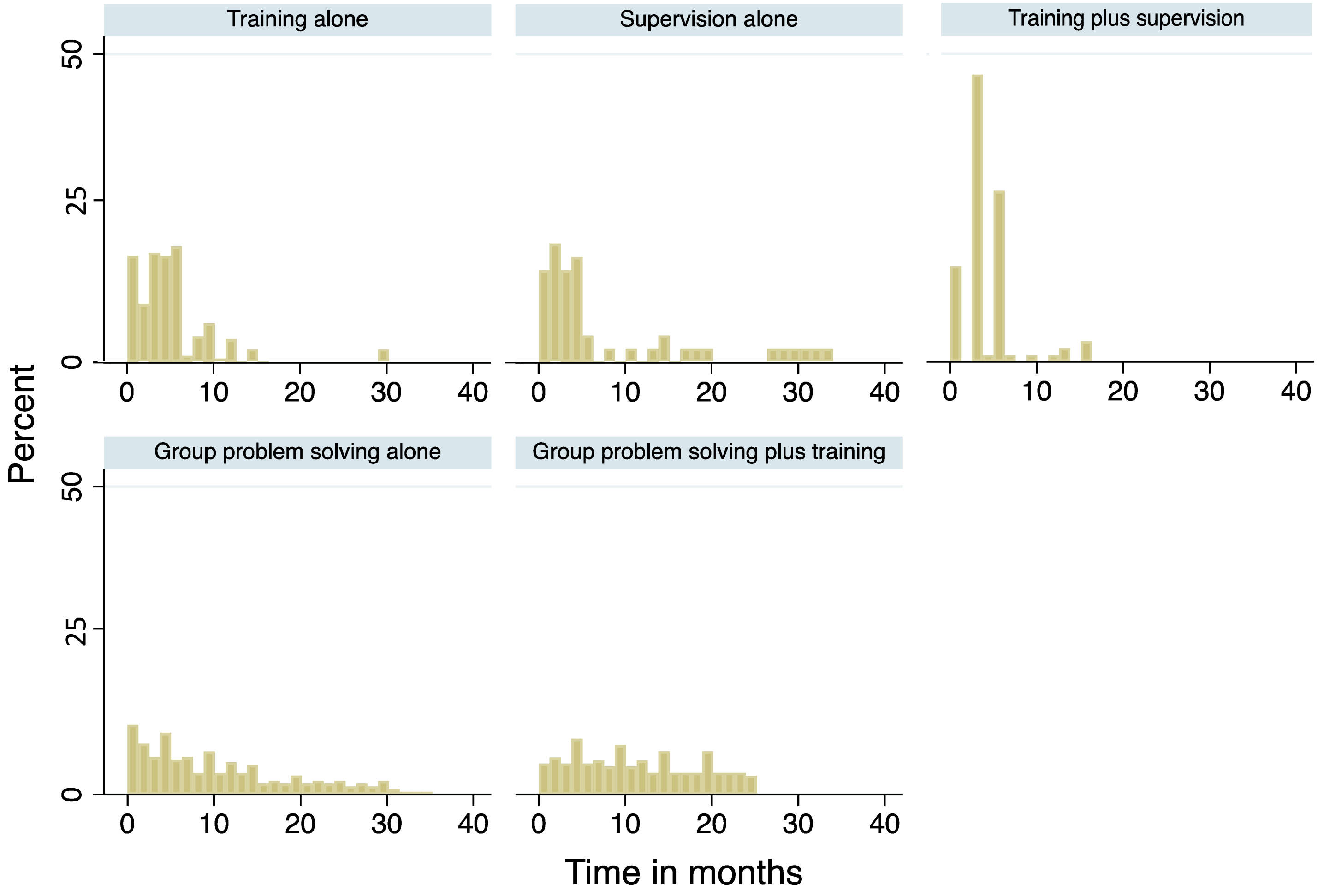

Supplement: Supplementary data [file bmjqs-2020-011717supp001.pdf]
